# Small molecule-mediated rapid maturation of human induced pluripotent stem cell-derived cardiomyocytes

**DOI:** 10.1186/s13287-022-03209-z

**Published:** 2022-12-27

**Authors:** Nino Chirico, Elise L. Kessler, Renée G. C. Maas, Juntao Fang, Jiabin Qin, Inge Dokter, Mark Daniels, Tomo Šarić, Klaus Neef, Jan-Willem Buikema, Zhiyong Lei, Pieter A. Doevendans, Joost P. G. Sluijter, Alain van Mil

**Affiliations:** 1 Circulatory Health Laboratory, Regenerative Medicine Center Utrecht, University Utrecht, University Medical Center Utrecht, Utrecht, The Netherlands; 2 Laboratory of Experimental Cardiology, Department of Cardiology, University Medical Center Utrecht, Utrecht, The Netherlands; 3 Center for Physiology and Pathophysiology, Institute for Neurophysiology, Faculty of Medicine and University Hospital Cologne, University of Cologne, Cologne, Germany; 4 Department of Cardiology, Amsterdam Medical Centre, 1105 AZ Amsterdam, The Netherlands; 5 Netherlands Heart Institute, Utrecht, The Netherlands

**Keywords:** Human induced pluripotent stem cell-derived cardiomyocyte, Maturation, Asiatic acid, GW501516, PGC-1α, PPAR

## Abstract

**Background:**

Human induced pluripotent stem cell (iPSC)-derived cardiomyocytes (iPSC-CMs) do not display all hallmarks of mature primary cardiomyocytes, especially the ability to use fatty acids (FA) as an energy source, containing high mitochondrial mass, presenting binucleation and increased DNA content per nuclei (polyploidism), and synchronized electrical conduction. This immaturity represents a bottleneck to their application in (1) disease modelling—as most cardiac (genetic) diseases have a middle-age onset—and (2) clinically relevant models, where integration and functional coupling are key. So far, several methods have been reported to enhance iPSC-CM maturation; however, these protocols are laborious, costly, and not easily scalable. Therefore, we developed a simple, low-cost, and rapid protocol to promote cardiomyocyte maturation using two small molecule activators of the peroxisome proliferator-activated receptor β/δ and gamma coactivator 1-alpha (PPAR/PGC-1α) pathway: asiatic acid (AA) and GW501516 (GW).

**Methods and Results:**

Monolayers of iPSC-CMs were incubated with AA or GW every other day for ten days resulting in increased expression of FA metabolism-related genes and markers for mitochondrial activity. AA-treated iPSC-CMs responsiveness to the mitochondrial respiratory chain inhibitors increased and exhibited higher flexibility in substrate utilization. Additionally, structural maturity improved after treatment as demonstrated by an increase in mRNA expression of sarcomeric-related genes and higher nuclear polyploidy in AA-treated samples. Furthermore, treatment led to increased ion channel gene expression and protein levels.

**Conclusions:**

Collectively, we developed a fast, easy, and economical method to induce iPSC-CMs maturation via PPAR/PGC-1α activation. Treatment with AA or GW led to increased metabolic, structural, functional, and electrophysiological maturation, evaluated using a multiparametric quality assessment.

**Supplementary Information:**

The online version contains supplementary material available at 10.1186/s13287-022-03209-z.

## Introduction

Remarkable progress has been made over the past decade in the differentiation of human induced pluripotent stem cells (iPSCs) into functional iPSC-derived cardiomyocytes (iPSC-CMs). However, the resulting human iPSC-CMs are not fully comparable to their adult primary counterparts in terms of metabolism, structure, function, and electrophysiology [1]. Rather, they bear a strong resemblance to immature cardiomyocytes seen in the late foetal stage. This limits their applications, as most (genetic) cardiac diseases do not occur until middle-age [2–5]. Recently, maturation of iPSC-CMs through different approaches has gained traction, including prolonged time in culture [6, 7], use of specialized medium [8–11], activating specific metabolic pathways, electrical and/or mechanical stimulation, and encapsulation in a 3D environment [12–15]. Future progress will potentially come from identifying and mimicking developmental drivers [16]. During foetal development, cardiomyocytes show an extensive increase in contractile cytoskeleton protein content [17] and undergo ion channel remodelling [18]. Between the foetal stage and early adolescence, human cardiomyocytes undergo hypertrophy with an increase in myofibril mass, with cell sizes increasing 10- to 20-fold, and loss of self-depolarization outside the nodal population [19]. Furthermore, cardiomyocytes undergo remodelling of intercellular junctions, T-tubule formation, and increase in DNA content leading to polyploidy in a single nucleus or binucleation in about 60% of cells [19–21]. Cardiomyocytes experience an extensive increase in energetic demands leading to increased mitochondrial mass and structural changes, and a switch from anaerobic glycolysis to oxidative phosphorylation, in particular fatty acid (FA) oxidation (FAO) [22, 23]. Long-chain FAO produces 3–4 times more ATP per molecule compared to glucose oxidation, thus increasing energy supply, but at the cost of increased oxygen consumption [24].

**Fig. 1:**
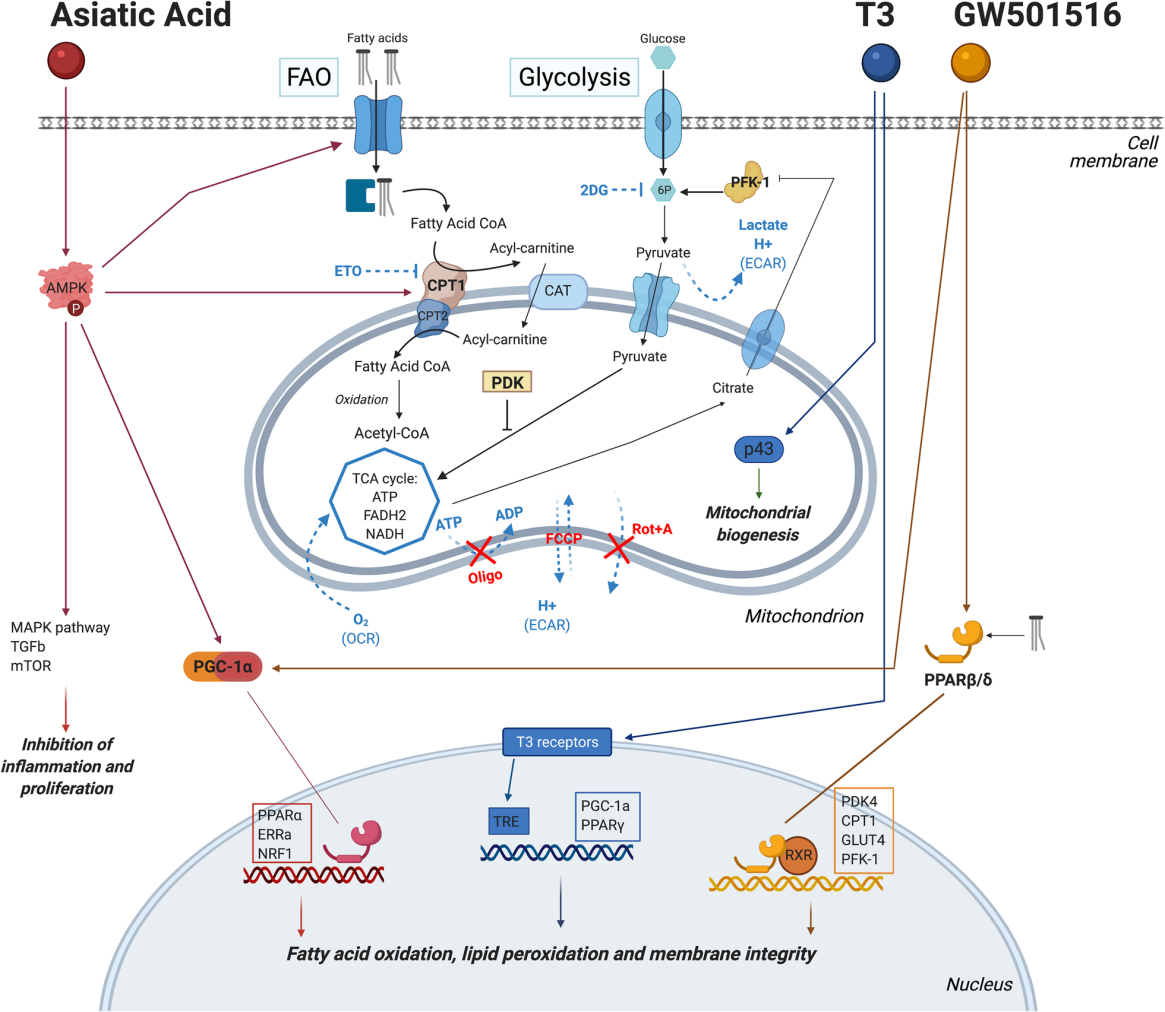
Schematic overview of the main cellular metabolic pathways with the proposed action mechanisms of small molecules asiatic acid and GW501516, as well as T3 hormone that was used as a positive control

We hypothesized that, next to providing the cells with the proper metabolic substrate—FA—a short treatment activating the peroxisome proliferator-activated receptor β/δ and gamma coactivator 1-alpha (PPAR/PGC-1α) pathway via the addition of small molecules could trigger FAO metabolism and improve cell maturation. Hence, we selected two small molecules—asiatic acid (AA) and GW501516—to specifically activate the PPAR/PGC-1α pathway in iPSC-CMs. T3 is a hormone involved in cardiac development, well known for its effect on iPSC-CMs maturation and has been shown to stimulate mitochondrial respiratory capacity and biogenesis, by activating p43, an activator of mitochondrial genome replication [11, 25–27]. In this study, T3 was used as a positive control due to its known increased respiratory capacity and electrophysiological maturation in iPSC-CMs, though the mechanism has not been established yet [28] (Fig. 1).

### Asiatic acid and GW501516

Asiatic acid (AA; Fig. 1) is a naturally occurring pentacyclic triterpenoid, which is attributed a wide spectrum of biological activities [29–31]. In the context of metabolism, AA increases the activity of enzymes involved in lipid synthesis like 3-hydroxy-3-methyl-glutaryl-CoA-reductase and metabolic regulators like adenosine monophosphate-activated protein kinase (AMPK) [31]. AMPK regulates the switch between glycolytic and oxidative metabolism by controlling the FA availability via increased FA plasma membrane uptake and utilization. Furthermore, AMPK promotes FA entry into the mitochondria and β-oxidation via carnitine palmitoyl transferase (CPT1) [32]. In addition, AMPK activates PGC-1α, which in combination with peroxisome proliferator-activated receptors is responsible for the long-term stimulation of FAO in skeletal muscle and the heart [24, 33–38]. AA is also able to reduce oxidative stress and apoptosis via alpha-synuclein (α-syn) entry inhibition and release of cytochrome c from the mitochondria. Pre-treatment with AA was shown to increase peroxisome proliferator-activated receptor-gamma coactivator 1-alpha (PGC-1α) levels, enhancing mitochondrial biogenesis [39–41]. GW501516 (GW; Fig. 1) is a synthetic, highly selective agonist of PPARβ/δ, which is the most prevalent PPAR isoform in the heart and responsible for cardiac FA metabolism regulation and lipid utilization in muscle tissues [24, 34, 37, 38, 42–44].

In this study, we show that after ten days of treatment with a single substance, both AA and GW triggered a metabolic switch and induced maturation of iPSC-CMs, providing a rapid and cost-effective method to obtain iPSC-CMs that more closely resemble their adult primary counterparts.

## Materials and methods

### Human iPSC culture and differentiation

**Fig. 2:**
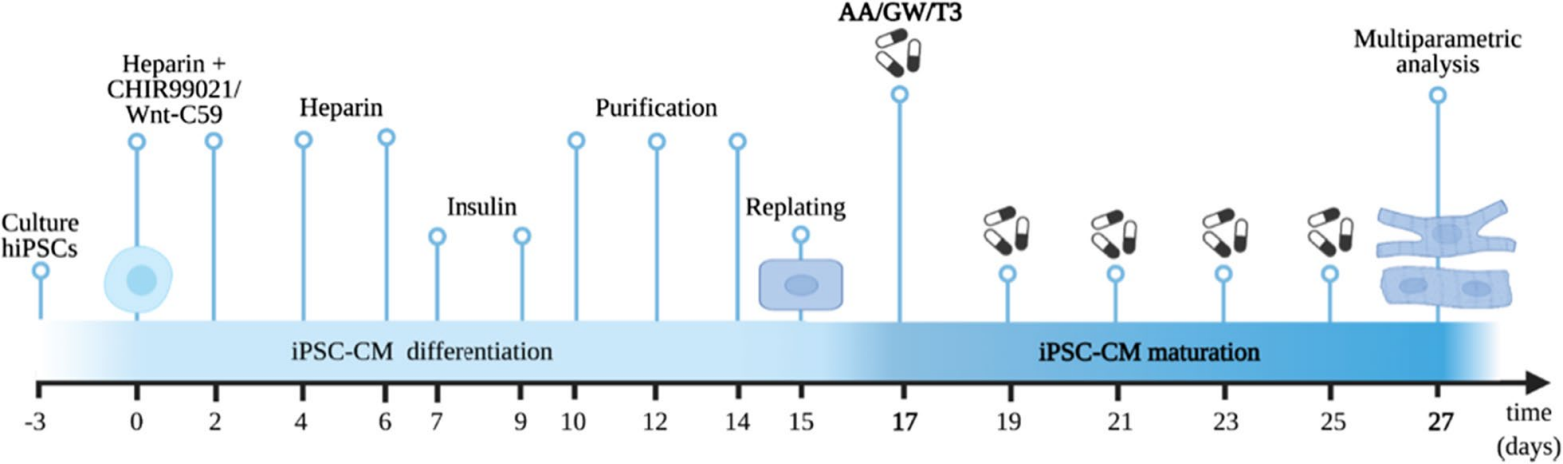
Schematic representation of timeline and experimental setup as described in the “Materials and methods” section. Differentiated iPSC-CMs were replated on day 15 and treated with the selected small molecules every other day until day 27

Three healthy Caucasian fully characterized human iPSCs were generated from peripheral blood mononuclear cells by using the insertion-free Sendai virus reprogramming method: UKKi032-C (NP0141-31B) (P34–P49) [45] and UKKi036-C (NP0143-18) (P26–P47), females and UKKi037-C (NP0144-41, male) (P29–P36). All these cell lines have been deposited in the European Bank for induced pluripotent Stem Cells (EBiSC, https://ebisc.org/) and are registered in the online registry for human PSC lines hPSCreg (https://hpscreg.eu/). All experiments were conducted according to the criteria of the code of proper use of human tissue used in the Netherlands. iPSCs were cultured on growth‐factor‐reduced Matrigel (Corning) in Essential 8™ medium (Gibco A1517001) that was changed daily. Cells were non-enzymatically passaged every 4–5 days with 0.5 × 10^−3^ M EDTA (Thermo Fisher Scientific). We differentiated iPSCs to CMs using a GiWi differentiation protocol adapted from [46] (Fig. 2). In detail, at day 0 of the differentiation, with iPSCs at 85% confluency, medium was changed to heparin medium (see Additional file 1: Table S1 for medium composition) with 4 × 10^−6^ M CHIR99021 (Selleck Chemicals). After 48 h, medium was replaced with heparin medium containing 2 × 10^−6^ M Wnt‐C59 (Tocris Bioscience). At day 4 and 6, medium was replaced with heparin medium. At day 7, medium was changed every other day to insulin medium (Additional file 1: Table S1) until purification at day 10. iPSC-CMs were beating around day 10, and medium was changed to purification medium (Additional file 1: Table S1) until day 15. All cultures were routinely tested for mycoplasma contamination using a MycoAlert Kit (Lonza). In the differentiation process, we included a purification and a replating step to generate a high-purity cardiomyocyte population (Average 91.5% ± 9.5 ACTN + ; Additional file 1: Fig. S1).

### Treatment with small compounds

At day 15, iPSC‐CMs were replated using TrypLE Select Enzyme 10X (Gibco) at 5 × 10^6^ cells/ 24-well in replating medium (Additional file 1: Table S1). At day 17, medium was replaced with basal maturation medium (Additional file 1: Table S1) supplemented with a selected concentration of asiatic acid (Selleck Chemicals, stock concentration 10 mM in dimethyl sulfoxide, DMSO), GW501516 (Enzo Life Sciences, stock concentration 1 mM in DMSO), or 3,3ʹ-triiodo-I-thyronine (T3) hormone as a positive control for iPSC-CMs maturation (Sigma-Aldrich, stock concentration 1 mM in DMSO). Basal maturation medium supplemented with 1:1000 dilution of DMSO was used as negative control (CTRL). Medium supplemented with fresh substances was changed every other day until day 27.

### Quantitative real‐time polymerase chain reaction (qRT‐PCR)

Total RNA was isolated with TriPure Isolation Reagent (Roche Applied Science) and treated with RNase‐free DNase I (Qiagen). In total, 500 ng DNA‐free RNA was transcribed into cDNA using the iScript cDNA Synthesis Kit (Bio‐Rad). RT‐qPCR was performed using iQ SYBR Green Supermix (Bio‐Rad) with specific primers in a CFX96 Touch Real‐Time PCR detection system (Bio‐Rad): 5 min at 95 °C, followed by 40 cycles of 15 s at 95 °C, 30 s at specific annealing temperature, and 45 s at 72 °C, followed by melting curve analysis to confirm single product amplification. Messenger RNA (mRNA) expression levels were normalized to ribosomal protein L32 (*RPL32*) reference gene mRNA expression (ΔCt). Relative differences were calculated (ΔΔCt) and presented as fold induction (2^−ΔΔCt^). Primers used are shown in Additional file 1: Table S2. Data from three different iPSC-CMs lines and multiple differentiation batches were combined using a factor correction method as described in Ruijter et al. [47].

### Western blot

For immunoblotting, iPSC-CMs were scraped and lysed in RIPA lysis buffer (ThermoFisher) supplemented with protease inhibitors (PhosSTOP and cOmplete, Roche). All protein samples were separated on a gradient 12–4% SDS-PAGE gel, electro-transferred on nitrocellulose membranes, and blocked with 5% bovine serum albumin. Equal efficiency of protein transfer was assessed by Ponceau-S staining. Membranes were incubated with mouse monoclonal antibodies against Connexin-43 and L-type Calcium channel Cav1.2. Secondary labelling was performed with an HRP-conjugated anti-mouse whole IgG antibody and detection was performed using standard ECL procedure with ChemiDoc XRS system (BioRad Laboratories). Ponceau-S staining as loading control to ensure uniform protein loading. Quantification was performed with ImageLab 6.1 (BioRad Laboratories 2020), where the protein of interest was corrected for the corresponding area from the Ponceau-S staining.

### Seahorse metabolic assay

Metabolic changes were quantified in iPSC-CMs via mitochondrial oxidation and glycolysis, which are evaluated by analysis of oxygen consumption rate (OCR, pmol/min) and extracellular acidification rate (ECAR, mpH/min) using a Seahorse XFe24 Extracellular Flux Analyzer (Seahorse Bioscience) in XFe24 microplates. In short, 15-day-old iPSC-CMs were seeded onto Matrigel-coated Seahorse XFe24 assay plates at a density of 5 × 10^5^ cells/well, allowed to adhere for two days, and thereafter cultured in either CTRL medium or supplemented maturation media (AA, GW, or AA + GW); replacing 200 µL medium every other day for ten days before the bioenergetic assay was performed, iPSC-CMs were washed three times with fresh Seahorse medium (Additional file 1: Table S1) and incubated for one hour in a non-CO_2_ incubator at 37 °C. Raw values (OCR and ECAR) were first normalized to cell nuclei count per well by Hoechst staining and 20X magnification imaging using the Evos microscope and ImageJ.

#### Mitochondria stress test

Following manufacture instructions, stress test inhibitors were sequentially added: oligomycin (2.5 mM), FCCP (2.5 mM), rotenone and antimycin A (Rot + A) (2.5 mM). Three measurements were taken before and after each injection and mixing cycle. ECAR and OCR were normalized to the last measurement of basal respiration before oligomycin was added (red arrow Fig. 4a). Baseline respiration, ATP production, proton leak, maximal respiration, and non-mitochondria respiration were calculated according to manufacture guidelines.

#### Fatty acid oxidation and glycolysis dependency

Substrate dependency was measured using a protocol adapted from Chou et al. [48]. Briefly, 100 µM of etomoxir (ETO Agilent) was added to irreversibly inhibit CPT1, followed by addition of 50 mM 2-Deoxy-d-glucose (2DG, Sigma-Aldrich) a competitive glycolytic inhibitor. FAO and glycolysis fluxes were measured by the OCR and ECAR, respectively [49]. Three measurements were taken before and after each injection and mixing cycle. ECAR values were normalized to the last measurement of basal respiration before ETO injection (red arrow); FAO dependency was calculated as the difference between basal OCR (after ETO addition), divided by the mitochondrial function from other substrates oxidation (after 2DG).

### Immunofluorescent labelling

For immunofluorescent labelling experiments, iPSC-CMs were seeded on coverslips (2.5 × 10^6^ cells/cm^2^) and fixated using 4% paraformaldehyde. Cells were permeabilized using 0.1% Triton‐X‐100 (Sigma‐Aldrich) for 10 min and blocked with 10% normal goat serum (Sigma‐Aldrich) for 30 min and then incubated at 4 °C overnight with primary antibodies (see Additional file 1: Table S3) diluted in DPBS. Secondary labelling was achieved by appropriate goat anti-mouse Alexa fluor‐488, and goat anti-rabbit Alexa fluor‐568 antibodies (Thermo Fisher Scientific, 1:500), and 1 µg/mL Hoechst (Thermo Fisher Scientific) for 4 h at room temperature. Images were taken using a Leica SP8X confocal microscope.

### Flow cytometry

To assess DNA content of a single nuclei, freshly collected iPSC-CMs (2 × 10^4^) were washed with PBS and fixated in 70% cold ethanol for 30 min. Cells were stained with propidium iodide solution (5 μg/mL propidium iodide and 250 μg/mL RNase in PBS) and analysed using Cytoflex flow cytometer #A00-1–1102 (Beckman Coulter).

### Optical calcium transient analysis and beating rate

Ca^2+^ transient analysis was performed to evaluate the Ca^2+^ handling function between CTRL, AA-, and GW-treated hiPSC-CMs monolayers. Briefly, cells were incubated 30 min in FluoroBrite DMEM Media (Thermo Fisher) supplemented with 1.25 µM Cal-520 (Abcam) and 0.02% Pluronic F-127 solution (Sigma-Aldrich). 30 s at 33 frames per second (fps) videos were automatically scanned by a Leica Thunder microscope. Analysis was conducted using Cyteseer (Vala Sciences, California, USA) as previously described [50]. The physiological parameters: rise time, calcium transient duration (CTD) at 25 and 75 percent, full width at half maximum (FWHM) representing 50% of the peak width, decay time, and beats per minute were automatically calculated for each time series. Data tables were analysed with Microsoft Excel and drug responses and bar plots were generated with GraphPad Prism 9 software.

Videos of monolayers of iPSC-CMs were taken using a GoPro Black Hero 7 camera connected to a bright field microscope via c-mount system. A 20 s video was taken at endpoint (D27), and beating rate (beats per minute, bpm) was manually calculated.

### Statistics

All data are expressed as mean ± standard error of the mean (SEM). Statistical analysis for not normally distributed qPCR data was performed using a nonparametric Wilcoxon signed-rank test. qPCR data are presented as control (CTRL) group versus treated groups (AA, GW, T3): median (25th—75th percentile; p-value) in the text and in the graphs. For the control group, in the graphs the mean is shown for clarity. For mitochondrial stress test and flex test, an Ordinary one-way ANOVA test was used. Flow cytometry data were analysed using a two-way ANOVA. All analyses were performed using GraphPad Prism 9.0.1 (GraphPad Software Inc., La Jolla, USA). A value of *p* < 0.05 was considered statistically significant.

## Results

### AA and GW treatment induced a switch to fatty acid metabolism

#### AA and GW optimal dosing to induce maturation-related gene expression is iPSC line-dependent

To determine optimal small molecule dosing to induce maturation, three iPSC lines were exposed to a wide range of AA, GW, and T3 concentrations according to the scheme shown in Fig. 2, and the optimal drug doses were selected based on their ability to increase the expression of the maturation-related carnitine palmitoyl transferase 1B (*CPT1B*) and cardiac troponin I (*TNNI3*) genes (Additional file 1: Fig. S2). CPT1B is a key mitochondrial enzyme that facilitates the transport of FAs into the mitochondrial matrix to enable β-oxidation, and TNNI3 is the main adult troponin of the cardiac sarcomeres. These titration experiments revealed an iPSC line-dependent dose–response effect on CMs at day 27 of differentiation (Fig. 2). For further experiments, the cell clone-dependent optimal concentrations (UKKi036-C: AA 5 and 2 μM, GW: 250 and 100 nM; and T3: 200, and 100 nM; UKKi032-C: AA 10 and 5 μM, GW: 1000 and 500 nM; and T3: 400, and 200 nM; UKKi037-C AA 10 and 5 μM, GW: 1000 and 500 nM; and T3 800 and 400 nM–Additional file 1: Fig. S2) were used.

#### AA and GW treatment increased iPSC-CM FA metabolism and mitochondrial function

**Fig. 3:**
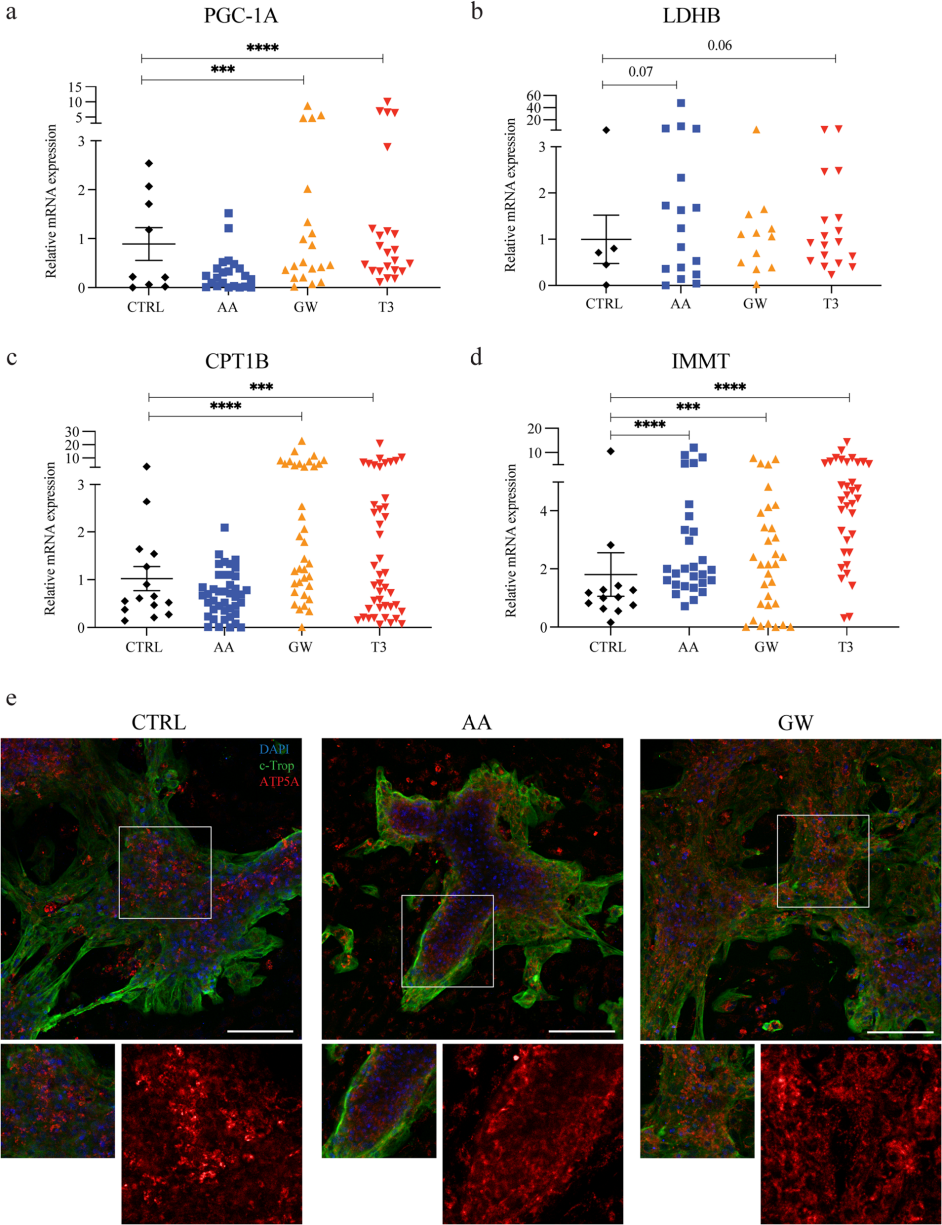
AA and GW increase iPSC-CM fatty acid metabolism and mitochondrial function gene expression. Combined relative gene expression of all three cell lines evaluated across multiple differentiation batches at D27. **a** *PGC1A* CTRL versus AA, GW, and T3, respectively: 0.22 (0.04–1.89) versus 0.22 (0.03–0.42, *n.s.*), 0.49 (0.25–1.85; *p* = 0.0005), and 0.71 (0.34–1.20; *p* < 0.0001), **b** *LDHB* CTRL versus AA, GW, and T3, respectively: 0.71 (0.23–1.91) versus 1.24 (0.3–3.90; *p* = 0.07), 1.09 (0.42–1.46, *n.s.*), and 0.93 (0.52–1.71; *p* = 0.06), **c** *CPT1B* CTRL versus AA, GW, and T3*,* respectively*:* 0.61 (0.37–1.54) versus 0.68 (0.33–1.08, *n.s.*), 1.62 (0.88–5.67; *p* < 0.0001), and 1.01 (0.40–2.68; *p* = 0.0008), and **d** *IMMT* CTRL versus AA, GW, and T3, respectively: 1.03 (0.69–1.35) versus 1.99 (1.45–3.70; *p* < 0.0001), 2.12 (0.77–3.81; *p* = 0.0005), and 4.43 (2.56–5.94; *p* < 0.0001). **e** Representative images of fluorescent double immunolabelling of iPSC-CMs (UKKi036-C D27) for cardiac troponin T (green) and ATP5A (red). Hoechst (blue) indicates nuclei. Scale bar: 100 µm

Physiologically, the formation and maintenance of a mitochondrial network allow intracellular distribution and synthesis of large amounts of ATP in mature cardiomyocytes [22, 51, 52]. First, the effect of treatment with AA, GW, and T3 on maturation-related metabolic changes in iPSC-CM, such as non-oxidative metabolism and mitochondrial function, FAO and lipid uptake, and mitochondrial biogenesis and function was assessed by RT-qPCR and immunostaining. RT-qPCR was performed to assess the mRNA expression levels of key regulators of cardiac metabolism *PGC1A* and lactate dehydrogenase B (*LDHB*) as well as markers for mitochondrial activity *CPT1B* and inner membrane mitochondrial protein (*IMMT*) (Fig. 3a–d). Expression of *PGC1A* was increased after GW and T3 treatment compared to untreated control (Fig. 3a). GW and T3 increased *PGC1A* expression: CTRL 0.22 (0.04–1.89), GW 0.49 (0.25–1.85; *p* = 0.0005) and T3 0.71 (0.34–1.20; *p* < 0.0001). Treatment with AA and T3 showed a trend towards increased *LDHB* expression levels as compared to CTRL, but this did not reach statistical significance. Relative expression level for CTRL was 0.71 (0.23–1.91) versus 1.24 for AA (0.3–3.90; *p* = 0.07) and 0.93 for T3 (0.52–1.71; *p* = 0.06) (Fig. 3b). Additionally, an increase in *CPT1B* indicated enhanced mitochondrial uptake of FAs in GW- and T3-treated conditions compared to CTRL: GW 1.62 (0.88–5.67; *p* < 0.0001) and T3 1.01 (0.40–2.68; *p* = 0.0008) versus CTRL 0.61 (0.37–1.54) (Fig. 3a-c). After treatment with either AA or GW, an approximate twofold upregulation of *IMMT*, encoding for a protein critical for adult cristae organization, was observed. This effect was similar to that exerted by T3. The relative expression of *IMMT* mRNA in CTRL versus AA, GW, and T3, respectively, was: 1.03 (0.69–1.35) versus 1.99 (1.45–3.70; *p* < 0.0001), 2.12 (0.77–3.81; *p* = 0.0005), and 4.43 (2.56–5.94; *p* < 0.0001) (Fig. 3d). iPSC-CMs were then immunolabeled with c-Troponin and ATP Synthase (ATP5A) to further validate how mitochondrial gene expression was matched with protein levels in treated iPSC-CMs (Fig. 3e). Furthermore, AA treatment increased expression of mitochondrial key enzymes. In particular mitochondrial electron transport chain, oxidative phosphorylation, and fatty acid β-oxidation-related genes (NADH: ubiquinone oxidoreductase subunit V3, NDUFV3, oxoglutarate dehydrogenase (OGDH), and cytochrome c oxidase COX3, COX5B) were highly expressed (Additional file 1: Fig. S3).

#### AA treatment improved the iPSC-CM response to mitochondrial stress and increased their metabolic substrate flexibility

**Fig. 4:**
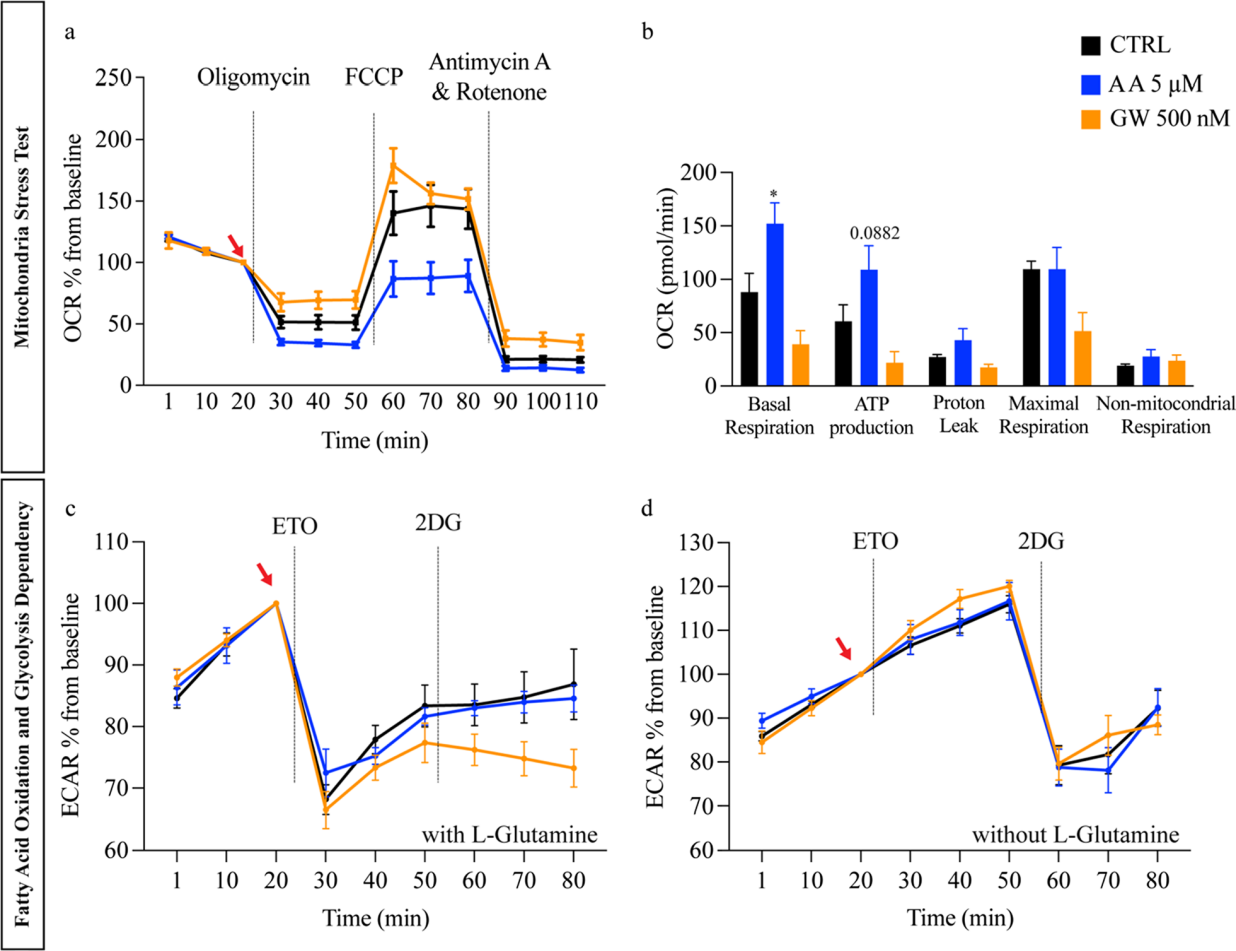
Effect of AA and GW on mitochondrial respiratory capacity and substrate flexibility of iPSC-CMs. Experiments were performed using D27 iPSC-CMs line UKKi036-C. **a** Relative oxygen consumption rate (OCR) in cells at day 27 of differentiation after sequential administration of oligomycin (ATP synthase inhibitor), FCCP (uncoupler of oxidative phosphorylation in mitochondria), and antimycin A and Rotenone (electron transport chain blockers) normalized to last value of basal respiration (baseline, red arrow). **b** OCR (pmol/min) for basal respiration (CTRL: 87.91 versus AA 152.07 and GW 39.25 *p* = 0.0217), ATP production (CTRL: 60.63 versus AA 109.03 and GW 14.7 *p* = 0.0882), proton leak, maximal respiration, and non-mitochondrial respiration. Ordinary one-way ANOVA was used to evaluate statical significance. **c** Relative extracellular acidification rate (ECAR) normalized to baseline (red arrow) of iPSC-CMs cultured in the Seahorse medium with L-glutamine. Addition of ETO and 2DG block the FAO and glucose-dependent glycolysis, respectively. **d** Relative ECAR normalized to baseline (red arrow) of iPSC-CMs cultured in the Seahorse medium without L-glutamine

After demonstrating enhanced gene and protein expression upon treatment with AA or GW, potential functional improvement of mitochondrial bioenergetics induced by the observed increased metabolic and mitochondrial gene expression was assessed by a Cell Mito Stress Test (Seahorse XF24 Extracellular Flux Analyzer). When adjusting the OCR to the basal respiratory rate (arrow Fig. 4a), the assay revealed that in response to oligomycin (ATP synthase inhibitor), the OCR value was approximately 60% lower in AA-treated samples (Fig. 4a). Furthermore, the assay revealed a twofold increase in basal mitochondrial respiration and ATP production in AA-treated iPSC-CMs compared to every other condition (*p* = *0.0217* Fig. 4b). The increase of glycolytic reserve (basal mitochondrial respiration after FCCP addition) indicated the enhanced capacity available to utilize glycolysis beyond the basal rate in the treated iPSC-CMs (Fig. 4a), even after glucose starvation during the maturation period (from D15 to D27).

Next, we investigated whether AA and GW enhanced FAO substrate utilization via a Seahorse flexibility test. The sequential administration of etomoxir (ETO) and 2-deoxyglucose (2DG) in this assay could identify substrate dependency. ETO is an inhibitor of CPT1 [53]—thus blocking the FA uptake and inhibiting FAO. 2DG is a hexokinase inhibitor, which inhibits the glucose-driven glycolytic pathway. GW-treated iPSC-CMs showed a decreased basal ECAR compared to CTRL, with AA showing a twofold increase (Additional file 1: Fig. S4a). The decrease in ECAR basal values indicate an accentuated FAO metabolism. The addition of ETO induced a fast approximately 30% decrease in ECAR for all conditions, with AA-treated iPSC-CMs exhibiting a faster recovery compared to CTRL (Fig. 4c). Higher ECAR recovery suggests that AA-treated iPSC-CMs have a higher capacity to switch between energetic pathways; from fatty acid β-oxidation to glycolysis, when the FA substrate is compromised. After the addition of 2-DG, OCR and ECAR values stayed constant, with a slow increase in AA-treated conditions (Additional file 1: Fig. S4b and Fig. 4c). This indicates that iPSC-CMs rely on l-glutamine (present in the Seahorse medium) as a metabolic substrate when both FAO and glycolysis are inhibited, since l-glutamine is not inhibited by 2-DG. Indeed, removing l-glutamine from the medium induced an approximately 50% decrease in ECAR values after 2-DG addition (Fig. 4d). These results suggest an increased ATP production via the TCA cycle (glutamine or lipid-dependent, according to their availability) in AA-treated conditions [54].

### AA and GW treatment enhanced contractile proteins expression, structural and ion channels gene expression, and calcium handling in iPSC-CMs

After demonstrating that AA and GW treatment increased metabolic maturation on a gene, protein, and functional level, other phenotypic hallmarks [2, 3] of structural, functional, and ion channel genes expression in cardiomyocyte maturation were evaluated: (i) increased mature sarcomere gene expression, (ii) increased DNA content per nuclei (polyploidism), and (ii) expression of repolarization-related ion channels.

#### AA and GW treatment improved sarcomeric-related gene expression

**Fig. 5:**
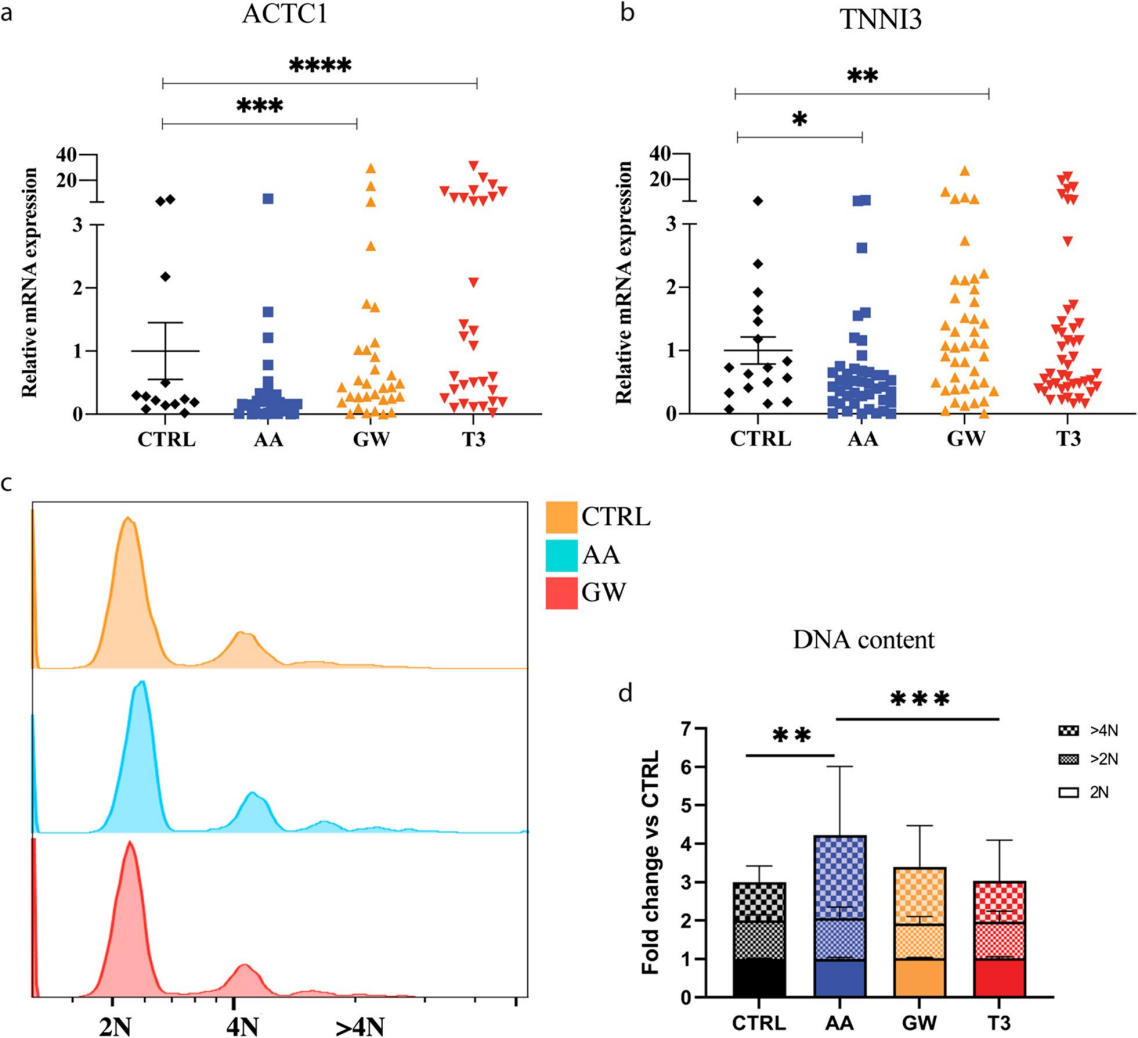
Small molecules improve iPSC-CM sarcomeric-related gene expression and polyploidy. The figures represent the combined relative gene expression of all three cell lines evaluated across multiple differentiation batches at D27. Relative sarcomeric gene expression **a** *ACTC1* in CTRL versus AA, GW, and T3, respectively: 0.24 (0.15–1.34) versus 0.16 (0.10 – 0.30; *n.s.*), 0.43 (0.24–1.02; *p* = 0.0008), and 1.08 (0.25–6.28; *p* < 0.0001); **b** *TNNI3* in CTRL versus AA, GW, and T3, respectively: 0.73 (0.37–1.55) versus 0.46 (0.2–0.68; *p* = 0.012), 1.08 (0.47–1.97; *p* = 0.002), and 0.63 (0.43–1.45; *p* = 0.09). **c**–**d** DNA content per single nucleus was measured using FACS on three independent experiments with iPSC lines: UKKi036-C, UKKi032-C, and UKKi037-C combined *n* = 6–12. c) Offset histogram of nuclei. d) DNA content per nuclei in treated iPSC-CMs normalized to CTRL for 2N, > 2N and > 4N. DNA content per nucleus > 4N CTRL 1, AA 2.16, GW 1.48, T3 1.06. *p*-values for polyploidism calculated via 2-way ANOVA ** = 0.0023, *** = 0.0003. Fig. S5 for more detail

Mature iPSC-CMs present organized, elongated, and dense sarcomeres with appropriate expression of Ca^2+^ pumps, ensuring physiological myocardial excitation and contraction [55]. GW and T3 treatment increased the expression of the major components of the contractile apparatus: GW induced a twofold increase in cardiac muscle α-actin 1 (*ACTC1*) mRNA expression, as compared to CTRL: GW 0.43 (0.24–1.02; *p* = 0.0008) versus CTRL 0.24 (0.15–1.34) while the expression of *TNNI3* mRNA was increased by approximately 1.5-fold: GW 1.08 (0.47–1.97; *p* = 0.002) versus CTRL 0.73 (0.37–1.55). No clear differences were observed upon AA treatment (Fig. 5a, b).

#### AA and GW treatment-induced iPSC-CM polyploidy

Studies have demonstrated that embryonic, foetal, and early postnatal cardiomyocytes can divide, whereas adult cardiomyocytes are predominately quiescent and, during early postnatal period, become binucleated in human [20, 56, 57]. After adolescence, the average DNA content per cardiomyocyte nucleus increases approximately 1.7-fold and remains constant with ageing [57, 58]. Therefore, cardiomyocyte polyploidy is considered an important hallmark in cardiomyocyte maturation. After treatment with AA, flow cytometry data showed an increase in tetraploidy compared to the CTRL and T3 group (2.16 versus 1.48-fold and 1.06, respectively) (Fig. 5c and Additional file 1: Table S4). This evidence combined with the absence of iPSC-CMs proliferation indicates an increased DNA content in the AA-treated samples (Additional file 1: Fig. S5).

#### AA and GW treatment enhances ion channel expression

**Fig. 6:**
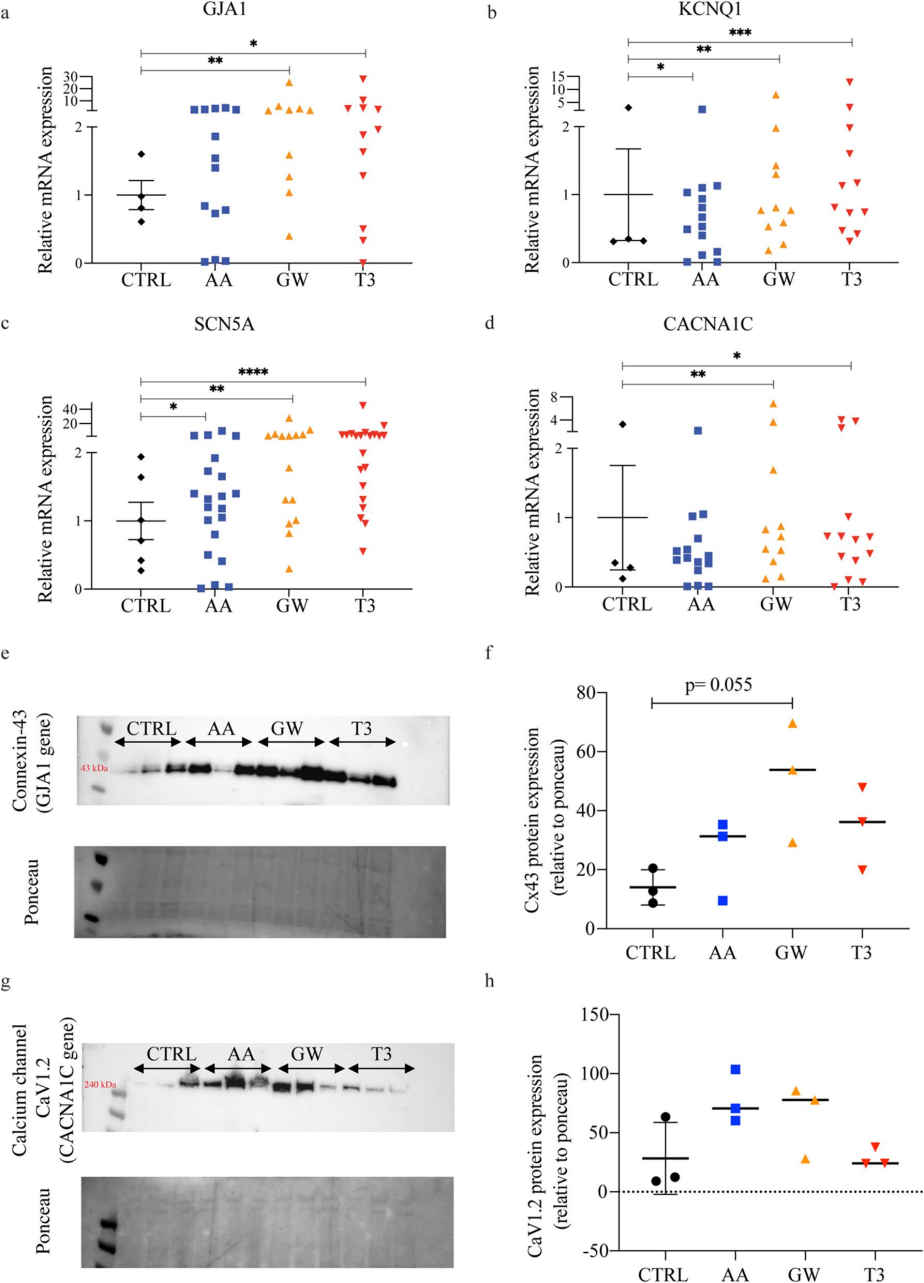
AA and GW treatment enhances ion channel expression. Relative gene expression of ion channel genes: **a** *GJA1* CTRL versus AA, GW, and T3, respectively: 0.90 (0.66–1.45) versus 1.50 (0.56–2.78; *n.s.*), 2.21 (1.21–4.15; *p* = 0.0098), and 1.92 (0.70–3.83; *p* = 0.021); **b** *KCNQ1* CTRL versus AA, GW, and T3, respectively: 0.34 (0.32–2.35) versus 0.6 (0.15–1.05; *p* = 0.048), 0.77 (0.53–1.43; *p* = 0.005), and 0.97 (0.54–0.89; *p* = 0.001); **c** *SCN5A* CTRL versus AA, GW, and T3, respectively: 0.86 (0.38–1.72) versus 1.32 (0.65–1.83; *p* = 0.034), 2.40 (1.01–4.49; *p* = 0.001), and 2.38 (1.41–4.07; *p* < 0.0001) **d** CACNA1C CTRL versus AA, GW, and T3, respectively: 0.32 (0.16–2.53) versus 0.42 (0.24–0.70; *n.s*.), 0.73 (0.37–1.69; *p* = 0.01), and 0.70 (0.31–1.41; *p* = 0.002). Western blots were performed on all three cell lines, and each lane contains pooled proteins from three experimental wells (total number of wells per condition = 9). Equal amount of protein was loaded in each lane; Ponceau staining is shown underneath the western blot panel as a loading control. **e** Cropped blots show protein levels of connexin-43 (43 kDa; *GJA1* gene), and **f** protein levels of calcium channel CaV1.2 (240 kDa; *CACNA1C* gene). Full-length blots are available in Fig. S6

Adult primary ventricular cardiomyocytes are electrically quiescent until triggered by the depolarization of a neighbouring cell, whereas immature iPSC-CMs beat spontaneously [59]. The expression of the mRNA encoding for the gap junction protein connexin 43 (*GJA1*), essential for a synchronized contraction, was increased over twofold after GW treatment (Fig. 6a). Western blot data confirmed an increase in protein content. In addition, even though voltage-gated K^+^ channels are essential for cardiac repolarization in mature cardiomyocytes, they are not all expressed in iPSC-CMs [18, 59, 60]. Here, iPSC-CMs treated with AA, GW, and T3 showed a two- and threefold increased expression in *KCNQ1* (Fig. 6b), a potassium channel involved in the termination of the cardiac action potential, normally not detectable in iPSC-CMs [18]. Additionally, the mRNA for Na^2+^ channel encoded by Na_v_1.5 α-subunit of the sodium channel (*SCN5A*) [61–63] was significantly upregulated upon AA, GW, and T3 treatment by, respectively, 1.5-fold, twofold, and threefold compared to CTRL (Fig. 6c). Expression of the mRNA encoding the L-type calcium channel (*CACNA1C*), responsible for the plateau phase typical of matured ventricular cardiomyocytes, was significantly increased over twofold in the GW- and T3-treated groups, whereas no difference was shown in AA (Fig. 6d). Western blot data confirm a twofold and threefold increase in connexin 43 content in AA- and GW-treated samples, respectively (Fig. 6e, f). Similarly, CACNA1C protein content in both AA and GW (albeit with cell-line variation) exhibits a threefold increase (Fig. 6 g, h). Furthermore, GW outperformed T3 in both connexin 43 and CACNA1C, whereases AA outperformed T3 only in CACNA1C content (Fig. 6f, h).

#### AA and GW improved calcium handling in unstimulated iPSC-CMs

**Fig. 7:**
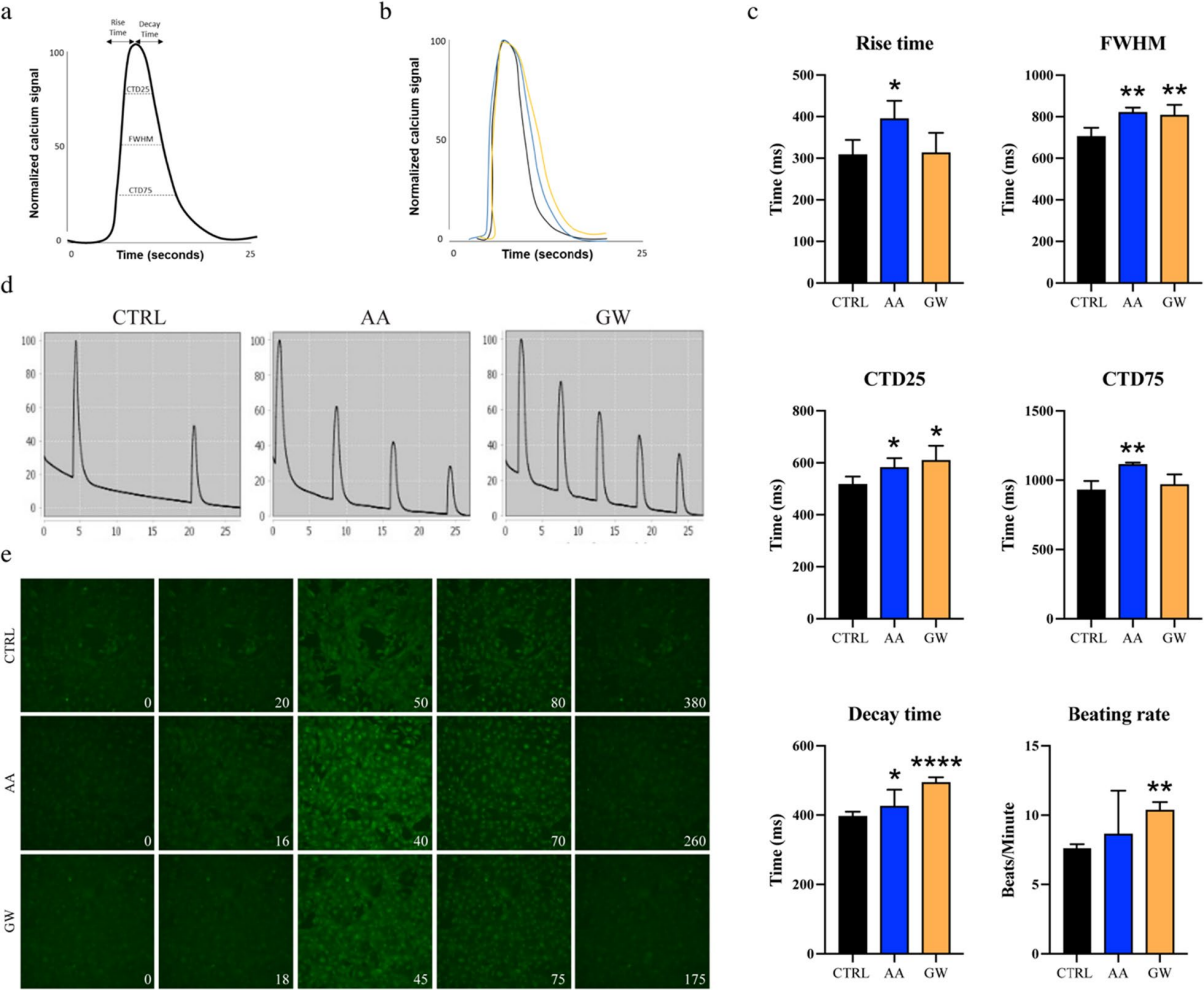
Measurements of calcium handling in unstimulated iPSC-CMs**.** **a** The calcium handling function was determined by calculating the rise time and decay time of the calcium signal and the duration of one contraction by the calcium transient duration (CTD) parameters. **b** Individual peak calcium signal normalized calcium signal over 30 s. **c** Rise time, durations at 25, 50, and 75 percentage of the maximum peak height, decay time, and beating rate values. **d** Normalized calcium signal over 30 s. **e** The calcium signal displays one calcium spark before the second spark in CTRL (380 frames), AA (260 frames) and GW (175 frames)

Following improved maturation of ion channel expression, we proceeded to analyse functionality in unstimulated iPSC-CMs after the ten days of AA or GW treatment (Fig. 7a). Measurements showed increased rise time, CTD25, FWHM, CTD75, and decay time in AA-treated samples (Fig. 7 b-e). Similarly, GW increased the CTD25, FWHM, decay time, and beating rate. (Fig. 7 b-e) These data overall indicate an improved calcium handling function and enlarged peak in the iPSC-CMs treated with AA or GW.

## Discussion

Nowadays, iPSC-CM differentiation protocols, in combination with the recently published expansion protocols, ensure a highly pure population of cardiomyocytes [64]. However, the created iPSC-CMs lack maturity. With this study, we propose a simple, economical, and fast method to induce iPSC-CM maturation via PPAR/PGC-1α activation in a FA supplemented medium.

**Fig. 8:**
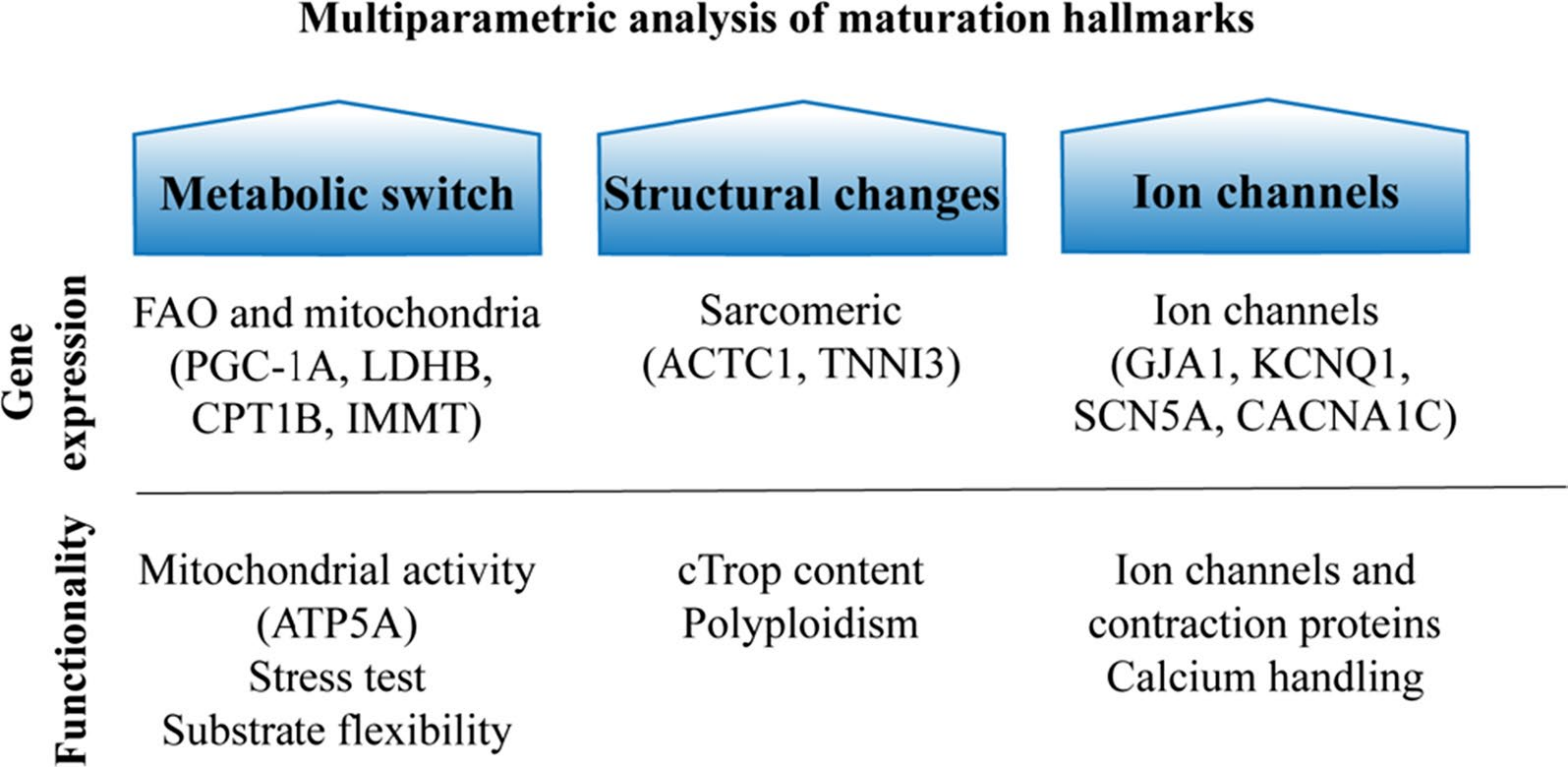
Workflow and readouts of this study

After ten days of treatment with AA, GW, or T3, iPSC-CM maturation, using three different cell lines, was evaluated using a multiparametric quality assessment, including metabolic gene expression, structural changes (sarcomere gene expression and polyploidy), and electrophysiology-related genes and ion channels protein expression (Fig. 8). We showed that FA metabolism and mitochondrial function were improved at the gene expression level (Fig. 3), and translated into enhanced metabolic activity, while maintaining their substrate flexibility (Fig. 4). Furthermore, contraction-related gene expression was enhanced (Fig. 5a, b), and iPSC-CM polyploidism (DNA content per single nucleus) was increased (Fig. 5c, d) after maturation with AA. Furthermore, an increase in specific ion channel protein content, gene expression, and calcium handling indicates enhanced maturation (Fig. 6 and 7). A simplified overview of the results is given in Table 1.

**Table 1:** Summary of single component effects on maturation main hallmarks

Component	Metabolism			Cellular structure		Electrophysiology	
	Mitochondria	FAO	Stress test	Contractile apparatus	Polyploidism	Ion channels	Contraction
AA	↑ organization ↑ ATP synthesis	↑ lactate metabolism ↑ recovery and substrate switch	↑↑ basal respiration ↑ ATP production	↑ sarcomere	↑ > 4N	↑ **gap junction** ↑ potassium ↑ sodium ↑ **calcium**	↑ rise time ↑ CTD25 ↑ FWHM ↑ CTD75 ↑ decay time
GW	↑ organization ↑ activity ↑ ATP synthesis	↑ FAO metabolism ↑ proton leak		↑↑ sarcomere		↑↑ **gap junction** ↑ potassium ↑ sodium ↑ **calcium**	↑ rise time ↑ CTD25 ↑ FWHM ↑ decay time
T3	↑ organization ↑ activity	↑ lactate metabolism ↑ FAO metabolism		↑ sarcomere		↑↑** gap junction** ↑ potassium ↑ sodium ↑ calcium	

Bold text indicates protein content

The two selected small molecules target the PPAR/PGC-1α pathway; GW has been shown to activate PPARβ/δ, thereby increasing PPARβ/δ/PGC-1α complexes, where PGC-1α functions as a coactivator that boosts PPARδ activation by direct protein–protein interaction [42–44, 65, 66]. In cardiomyocytes, PGC-1α activation has been linked to increased FA metabolism and mitochondrial biogenesis [34, 67]. PPARβ/δ plays a pivotal role in cardiac dysfunction, hypertrophy, and congestive heart failure via decreases in basal myocardial FAO [37, 42], whereas AA showed a protective effect against cardiac hypertrophy [31], suppressed mitochondria-mediated inflammasome activation [40], and reduced autophagy during ischaemia–reperfusion injury in a mouse model [29].

By replicating the adult cardiomyocyte metabolism, electrical and contractile characteristics, and physical appearance (e.g. multinucleation, sarcomere content) in iPSC-CMs, researchers are able to establish more clinically relevant human cardiac disease models and engineered tissues [68–70]. While the specific triggering mechanisms are not fully understood, cardiomyocyte maturation proceeds through concomitant structural, functional, and metabolic changes [3, 9, 10, 22, 70]. The latter occurs during foetal development when cardiomyocytes undergo a shift in their metabolism from glycolysis to FAO. In adult cardiomyocytes, FAO accounts for 80–90% of cell energy production compared to only 13% in foetal cardiomyocytes [21]. FAs constitute the main energy source necessary to support the high-energy demand of adult cardiomyocytes. Based on this, several groups demonstrated that a combination of galactose and FA leads to a glycolytic-oxidative metabolic shift and ultimately improving the iPSC-CM maturity [10, 71]. However, native mature cardiomyocytes maintain their metabolic flexibility allowing them to switch between different substrates, such as glucose, lactate, and glutamate [72]. In the present study, we directly targeted the FAO metabolism via GW or AA supplemented into the culture medium in the presence of FA. Activating the PGC1/PPAR pathway, resulted in an overall enhanced iPSC-CM maturation after only 10 days, thus indicating that cardiomyocyte maturation and FAO activation signalling events might be directly linked [25, 73, 74]. During maturation, the mitochondrial network of cardiomyocytes undergoes extensive remodelling to support the increased energy demand [75]. Kleiner et al. treated primary mouse myoblasts with 100 nM GW and demonstrated an increase in key FAO genes via PGC-1α; however, without effects on mitochondrial function [66]. Interestingly in our hands, GW increased both the expression of key regulators of cardiac metabolism and ATP5A content, indicating maturation of the mitochondrial network (Fig. 3). Previous findings suggested a neuroprotective effect of AA via mitochondrial biogenesis. Xu et al. showed how AA promotes a 1.5-fold increase in PGC-1α expression in vitro and restored lipid peroxidation in vivo [76]. Later studies confirmed that AA elevates the level of PGC1-α and increases the number of mitochondria, thus indicating an effect on mitochondrial biogenesis [39]. Concordant with our analysis a twofold increase in mitochondrial gene expression and FAO functionality was demonstrated, whereas no increase in PGC-1α expression was observed (Fig. 3). Previous evidence indicates PGC-1α as a central regulator of cardiac metabolism and promoter of oxidative phosphorylation at the expense of glycolysis [77, 78]. Whereas AMPK upregulation leads to improved mitochondrial activity and homeostasis in cardiomyocytes [79–82]. We found that AA-treated iPSC-CMs have undergone significant metabolic changes; genes responsible for the electron transport chain, oxidative phosphorylation, and fatty acid β-oxidation in mitochondria (*NDUFV3, COX3*, and *COX5B*) were highly expressed (Additional file 1: Fig. S3). These results are consistent with the changes in cellular fluxes (Seahorse) in AA-treated cells. Here, basal mitochondrial respiration, ATP production, and maximal respiration was increased indicating an increase in oxidative phosphorylation rate and in ATP synthesis (Fig. 4). Maximal respiration and ATP production represent the bioenergetic reserve of the cells [54]. Pour et al. explored cardiac mitochondrial transplantation to support high demand or acute/chronic stress. AA could potentially be used for a similar purpose, overcoming the issues connected to future invasive procedures such as mitochondrial transplantation [83]. In addition, treatment with oligomycin showed a limited effect on oxygen consumption in GW-treated cardiomyocytes, indicating that increased OCR aided a non-ATP producing purpose, possibly derived from proton leak, which has been shown by Zhang et al. to be naturally occurring in aged rodent cardiomyocytes [84]. In our study, maximal respiration could actually be dampened due to limits in ATP-dependent import of substrate (e.g. FA) in cardiomyocytes [54]. Taking together our Seahorse data indicated how AA treatment results in functional increase in respiration and ATP synthesis, and confirmed previous findings where AA was associated with reduced expression of FA synthesis genes and increased expression of FAO genes via AMPK and acetyl CoA carboxylase phosphorylation [85]. The healthy adult heart relies predominantly on FAs but can rapidly switch substrate preference depending on the physiological state (*e.g.* exercise) or pathologies. This metabolic flexibility is believed to be important for normal cardiac function [72]. The substrate flexibility test indicated that glucose-starved cardiomyocytes are dependent on specific energy substrates. The increase in glycolytic reserve indicates an enhanced capacity to utilize glycolysis beyond the basal rate in treated iPSC-CMs. It is of note that iPSC-CMs were cultured in glucose-free medium for ten days and when exposed to the Seahorse medium containing glucose, AA-treated cells’ high basal ECAR suggests an enhanced glycolytic process. AA-treated cardiomyocytes exhibiting a faster recovery and stronger capacity to switch from FAO to glycolysis when the fatty acid source was compromised. Both ECAR and OCR were unaffected by 2-DG (Fig. 4c), implying an increase in ATP production via the TCA cycle (glutamine-dependent) [54]. These data are linked to iPSC-CM maturation and comparable to previous research [73, 86]. When l-glutamine was removed from the Seahorse medium, it induced a sharp decrease in ECAR after 2-DG addition (Fig. 4d). This indicated that iPSC-CMs rely on l-glutamine (present in the Seahorse medium) as a metabolic substrate when both FAO and glycolysis are inhibited, since l-glutamine is not inhibited by 2-DG. Indeed, the use of l-Glutamine by cardiomyocytes has been associated with the regulation of FA entry and enhanced metabolism of long-chain FAO [87]. Overall, these findings indicate a unique role of fatty acid β-oxidation in regulating cardiomyocyte maturation and shows that AA and GW induce similar, but also complementary effects.

Furthermore, the small molecule treatment led to increased mRNA expression of contraction-related genes as well as ion channel protein content and gene expression. GW treatment resulted in an increased expression of connexin 43 (*GJA1*) and the Na_v_1.5 α-subunit of the sodium channel (*SCN5A*). Additionally, channels normally present only in adult cardiomyocytes were increased: KCNQ1*,* which is responsible for the action potential termination [18].

Additionally, we observed enhanced Ca^2+^ handling properties in iPSC-CMs after AA or GW treatment. The rise time, decay time, and beating rate improved in both conditions, indicating an increased calcium handling. The used parameters CD25, FWHM and CTD75 were also significantly increased in both the AA- and GW-treated iPSC-CM conditions, representing the width of the peak at each percentage measured from the peak in each waveform. Taken together, these results show that AA and GW can improve Ca^2^^+^ handling needed for the development of more forceful contraction, as seen in adult cardiomyocytes (Fig. 7).

Finally, in parallel with the enhanced ion channel expression and content, maturation of the contractile apparatus was shown by an increase in *ACTC1* and *TNNI3* expression (Fig. 5a, b). Importantly, treatment with AA induced a 2.16-fold increase in iPSC-CM tetraploidy (Fig. 5c), which aligns with Cao et al.’s report of a transient increase in DNA synthesis in maturing cardiomyocytes in mice [88]. Other reports show that AMPK and subsequently MAPK are both linked to cardiomyocytes multinucleation, however, only after activation upon oxidative stress or mitophagy [89–91]. AA is a known AMPK activator [31, 85] and, in our study, led to increased expression of CPT1 and cardiomyocyte increased polyploidism (DNA content per nuclei), without any detrimental effects on cardiomyocytes (Fig. 5c, d).

## Conclusions

Researchers are still far from generating phenotypical adultlike cardiomyocytes in vitro, and current protocols that enhance maturation are costly and laborious. Here, we presented a simple, scalable, and economical protocol to induce fast iPSC-CM maturation via addition of two small molecules (AA and GW) activating PGC1/PPAR pathways resulting in enhanced FA metabolism, mitochondrial function and higher metabolic activity, increased ion channel and sarcomere expression, and polyploidy, while maintaining substrate flexibility. Our developed method provides a tool that can be easily applied to improve iPSC-CMs maturity from metabolism and mitochondrial function, ion channel expression, to cellular structure and calcium handling, thus potentially helping advance cardiac disease modelling and cardiac regenerative approaches.

## Supplementary Information

**Additional file 1: Table S1**. Overview of culture Media used during this study. **Table S2.** Primer list used for qPCR experiments. **Table S3.** Antibodies list used for western blot, flow cytometry and fluorescent Immunohistochemistry experiments. **Fig. S2.** Cell line-dependent small molecules dose titration. iPSC line-dependent dose–response effect on CMs at day 27 of differentiation (Fig. 2). For further experiments, the cell clone-dependent optimal concentrations (UKKi036-C: AA 2 and 1 μM, GW: 250 and 100 nM; and T3: 200, and 100 nM; UKKi032-C: AA 10 and 5 μM, GW: 1000 and 500 nM; and T3: 400, and 200 nM; UKKi037-C AA 10 and 5 μM, GW: 1000 and 500 nM; and T3 800 and 400 nM – Fig. S2) were used. **Fig. S3.** AA and GW treatment enhances levels of mitochondrial key enzymes expression. a) OGDH CTRL versus AA, GW, and T3, respectively: 1.110 (0.6000 – 1.800) versus 1.919 (1.285–2.375; *p* = 0.0078) versus 2.608 (2.035–2.963. *n.s.*) versus 2.287 (1.520–2.770; *p *=0.0156); b) NDUFV3 CTRL versus AA, GW, and T3, respectively: 1.037 (0.7100–1.400;) versus 1.738 (1,553 – 1.933, *p* =0,0078), 3.750 (1.738 – 4.390; *n.s.*). and 2.251 (1.950 – 2.510; p=0.0156); c) COX3 CTRL versus AA. GW. and T3, respectively: 1.187 (0.4200–1.830) versus 1.166 (0.8725–1.433; *p* = 0.0234) versus 2.860 (1.918–3.753; *p* = *n.s.*) versus 2.203 (1.760–2.880; *p* = 0.0156); d) COX5 CTRL versus AA. GW. and T3, respectively: 1.053 (0.7800–1.540) versus 2.471 (1.950–2.943; *p* = 0.0078) versus 9.758 (6.838–13.52; *p* = *n.s.*) versus 2.873 (2.460–3.320; *p* = 0.0156). **Fig. S4.** Experiments were performed using day 27 iPSC-CMs line UKKi036-C. (a) Raw extracellular acidification rate (ECAR) of iPSC-CMs cultured in the Seahorse medium with L-glutamine. Addition of ETO and 2DG block the FAO and glucose-dependent glycolysis, respectively. (b) Raw oxygen consumption rate (OCR) in cells at day 27 of differentiation after sequential administration of ETO and 2DG. **Table S4.** Relative DNA content per single nucleus measured on three independent experiments with iPSC lines: UKKi036-C UKKi032-C, and UKKi037-C combined *n* = 6–12. **Fig. S5.** Cell nuclei count per well measured by Hoechst staining and 20X magnification imaging using the Evos microscope and ImageJ show no significant changes in number of nuclei present. **Fig. S6.** Full-length blots of connexin-43 (left) and CaV1.2 (right) with marker and molecular weight of protein of interest in red. Western blots were performed on pooled proteins from all three cell lines. Equal amount of protein was loaded in each lane. Ponceau staining is shown underneath the western blot panel as a loading control.

## Data Availability

The datasets generated during and/or analysed during the current study are not publicly available but are available from the corresponding author on reasonable request.

## Abbreviations

2DG: 2-Deoxy-d-glucose
AA: Asiatic acid
ACTC1: Actin alpha cardiac muscle 1
ADP: Adenosine diphosphate
AMPK: 5ʹ Adenosine monophosphate-activated protein kinase
ATP: Adenosine triphosphate
ATP5A: ATP synthase
CACNA1C: Calcium voltage-gated channel subunit alpha1 C
CAT: Carnitine acetyltransferases
CPT: Carnitine palmitoyltransferase
DMSO: Dimethyl sulfoxide
DNA: Deoxyribonucleic acid
ECAR: Extracellular acidification rate
ERRa: Estrogen-related receptor alpha
ETO: Etomoxir
FA: Fatty acid
FADH: Flavin adenine dinucleotide
FAO: Fatty acid oxidation
FCCP: Carbonyl cyanide P-(tri-fluromethoxy) phenyl-hydrazone
GJA1: Gap junction protein connexin 43
GLUT4: Glucose transporter 4
GW: GW501516
IMMT: Inner mitochondrial membrane protein
iPSC: Induced pluripotent stem cell
KCNQ1: Potassium voltage-gated channel subfamily Q member 1
LDHB: Lactate dehydrogenase B
MAPK: Mitogen-activated protein kinase
mTOR: Mammalian target of rapamycin
NADH: Nicotinamide adenine dinucleotide
NRF: Nuclear respiratory factor
OCR: Oxygen consumption rate
PDK: Pyruvate dehydrogenase kinase
PFK: Phosphofructokinase-1
PGC: Peroxisome proliferator-activated receptor-gamma coactivator
PPAR: Peroxisome proliferator-activated receptor β/δ
PPARGC1A/PGC-1α: Peroxisome proliferator-activated receptor-gamma coactivator 1-alpha
Rot + A: Rotenone and antimycin A
RPL32: Ribosomal protein L32
RXR: Retinoic acid receptors
SCN5A: Sodium voltage-gated channel alpha subunit 5
SERCA: Sarco/endoplasmic reticulum Ca2 + -ATPase
T3: 3,3ʹ-Triiodo-l-thyronine
TCA: Tricarboxylic acid cycle
TGFb: Transforming growth factor beta
TNNI3: Cardiac troponin I
α-syn: Alpha-synuclein
