# Supplementary material for: Small molecule-mediated rapid maturation of human induced pluripotent stem cell-derived cardiomyocytes
Source: Stem Cell Res Ther. 2022 Dec 27;13:531. doi: 10.1186/s13287-022-03209-z (PMC9795728; doi:10.1186/s13287-022-03209-z)
Supplement: Supplementary file 1 — Additional file 1: Table S1. Overview of culture Media used during this study. Table S2. Primer list used for qPCR experiments. Table S3. Antibodies list used for western blot, flow cytometry and fluorescent Immunohistochemistry experiments. Fig. S2. Cell line-dependent small molecules dose titration. iPSC line-dependent dose–response effect on CMs at day 27 of differentiation (Fig. 2). For further experiments, the cell clone-dependent optimal concentrations (UKKi036-C: AA 2 and 1 μM, GW: 250 and 100 nM; and T3: 200, and 100 nM; UKKi032-C: AA 10 and 5 μM, GW: 1000 and 500 nM; and T3: 400, and 200 nM; UKKi037-C AA 10 and 5 μM, GW: 1000 and 500 nM; and T3 800 and 400 nM – Fig. S2) were used. Fig. S3. AA and GW treatment enhances levels of mitochondrial key enzymes expression. a) OGDH CTRL versus AA, GW, and T3, respectively: 1.110 (0.6000 – 1.800) versus 1.919 (1.285–2.375; p = 0.0078) versus 2.608 (2.035–2.963. n.s.) versus 2.287 (1.520–2.770; p =0.0156); b) NDUFV3 CTRL versus AA, GW, and T3, respectively: 1.037 (0.7100–1.400;) versus 1.738 (1,553 – 1.933, p =0,0078), 3.750 (1.738 – 4.390; n.s.). and 2.251 (1.950 – 2.510; p=0.0156); c) COX3 CTRL versus AA. GW. and T3, respectively: 1.187 (0.4200–1.830) versus 1.166 (0.8725–1.433; p = 0.0234) versus 2.860 (1.918–3.753; p = n.s.) versus 2.203 (1.760–2.880; p = 0.0156); d) COX5 CTRL versus AA. GW. and T3, respectively: 1.053 (0.7800–1.540) versus 2.471 (1.950–2.943; p = 0.0078) versus 9.758 (6.838–13.52; p = n.s.) versus 2.873 (2.460–3.320; p = 0.0156). Fig. S4. Experiments were performed using day 27 iPSC-CMs line UKKi036-C. (a) Raw extracellular acidification rate (ECAR) of iPSC-CMs cultured in the Seahorse medium with L-glutamine. Addition of ETO and 2DG block the FAO and glucose-dependent glycolysis, respectively. (b) Raw oxygen consumption rate (OCR) in cells at day 27 of differentiation after sequential administration of ETO and 2DG. Table S4. Relative DNA content per single nucleus measured on three independent e [file 13287_2022_3209_MOESM1_ESM.docx]

# Supplementary Information


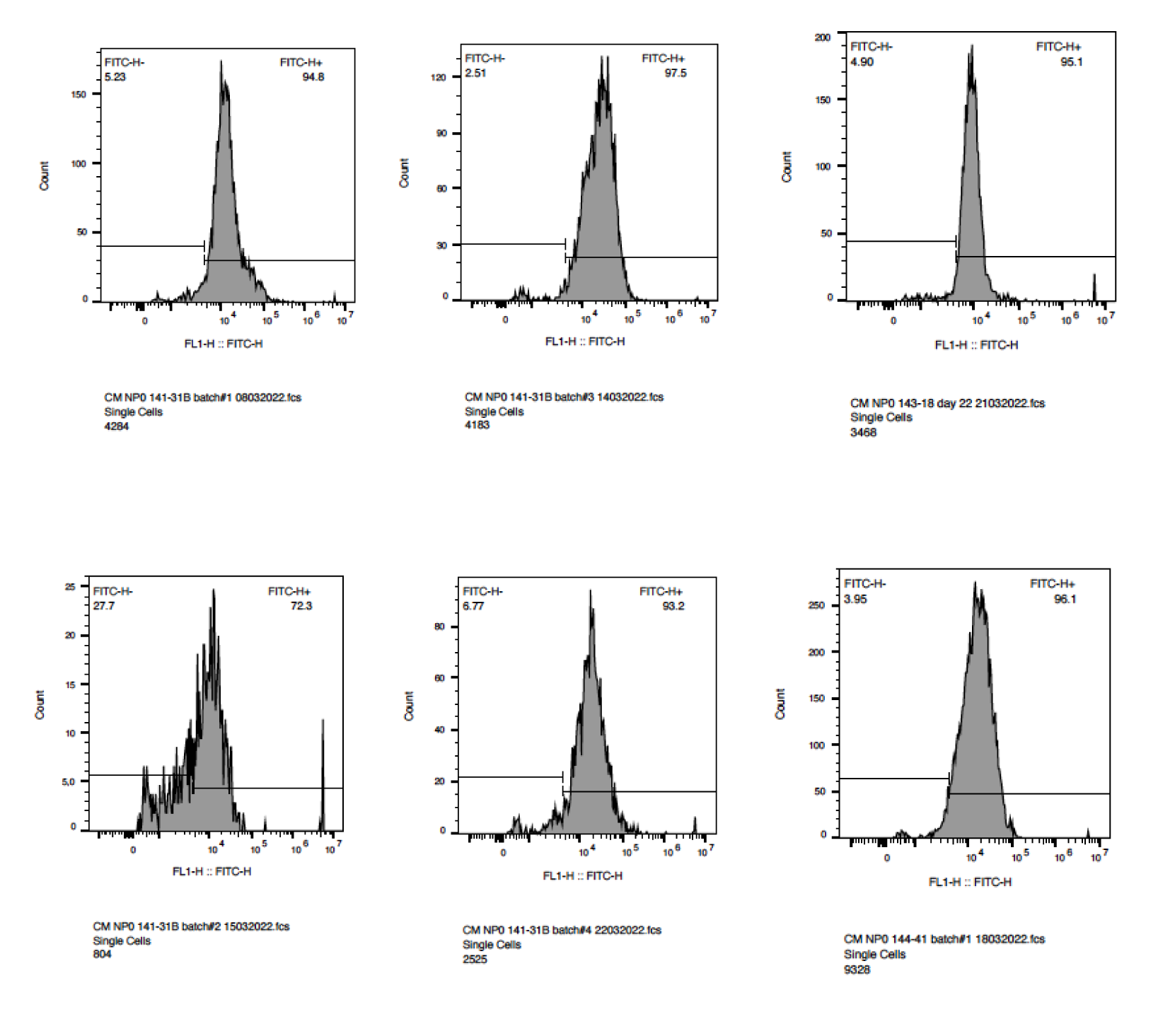


**Figure S1:** FACS plot shows a representative differentiation of three lines of iPSC-CMs with a high-purity cardiomyocyte population (Average 91,5% ­±9,5 ACTN+).

**Table S1:** Overview of culture Media used during this study.

| **Name** | **Media** | **Supplements** |
| --- | --- | --- |
| Heparin medium (HM) | DMEM-F12 50/50 (Thermo Fisher Scientific, 31331) | 213 μg mL-1 L-ascorbic acid 2 phosphate (Sigma-Aldrich, A8960-5G)  1:100 Chemically defined lipid concentrate (Thermo Fisher Scientific, 11905031)  1.5 IU mL-1 Heparin (Leo Pharmaceuticals Ltd.)  1% Penicillin-Streptomycin (Gibco, 15-140-122) |
| Insulin medium (IM) | DMEM-F12 50/50 (Thermo Fisher Scientific, 31331) | 213 μg mL-1 L-ascorbic acid 2 phosphate (Sigma-Aldrich, A8960-5G)  1:100 Chemically defined lipid concentrate (Thermo Fisher Scientific, 11905031)  21 µg mL-1 Human recombinant insulin (Sigma-Aldrich, I9278-5ML)  1% Penicillin/streptomycin (Gibco, 15-140-122) |
| Purification medium (PM) | RPMI 1640 L-Glutamine without glucose (Gibco, 11879) | 3.5 μM Sodium-dl-Lactate (Sigma-Aldrich, L4263)  213 μg mL-1 L-ascorbic acid 2 phosphate (Sigma-Aldrich, A8960-5G)  1:100 Chemically defined lipid concentrate (Thermo Fisher Scientific 11905031)  21 µg mL-1 Human recombinant insulin (Sigma-Aldrich I9278-5ML)  1% Penicillin/streptomycin (Gibco 15-140-122) |
| Replating medium (RM) | RPMI 1640 L-Glutamine without glucose (Gibco, 11879) | 5 µM ROCK inhibitor (Sigma-Aldrich, Y0503)  10% KnockOutTM serum replacement (KOSR) (Thermo Fisher Scientific, 10828028)  1% B27® (Gibco, 17504-001)  1% Penicillin/streptomycin (Gibco 15-140-122) |
| iPSC culture medium | Essential 8^TM^ medium (Gibco A1517001) |  |
| Basal maturation medium | DMEM, no glucose (Gibco, Thermo Fisher Scientific 11966025, USA) | 1:100 Chemically defined lipid concentrate (Thermo Fisher Scientific 11905031)  10 mM Galactose (Sigma-Aldrich G0750)1% Penicillin-Streptomycin (Gibco, 15-140-122) |
| Seahorse culture media | Agilent Technologies/Seahorse Bioscience) | 1% Chemically Defined Lipid Concentrate (Gibco),  4mM L-glutamine (Gibco)  10mM glucose (Agilent, 103577-100) |

Table S2: Primer list used for qPCR experiments.

| **Target gene** | **Primer name** | **Sequence (5'-3')** |
| --- | --- | --- |
| Carnitine Palmitoyltransferase 1B | CPT1B F | CTCCTTTCCTTGCTGAGGTG |
|  | CPT1B R | TCTCGCCTGCAATCATGTAG |
| Peroxisome Proliferator-Activated Receptor Gamma Coactivator 1-Alpha | PPARGC1A F | GCTTTCTGGGTGGACTCAAGT |
|  | PPARGC1A R | GAGGGCAATCCGTCTTCATCC |
| Lactate Dehydrogenase B | LDHB F | TCTGTGACCGCCAATTCTAAGA |
|  | LDHB R | GCACCAGATTGAGCCGACTC |
| Inner Mitochondrial Membrane Protein | IMMT F | GTCCTCCGTCCATTGCGAC |
|  | IMMT R | GGACCAAGAACCATCTCGAAG |
| Cardiac Troponin I | TNNI3 F | TTTGACCTTCGAGGCAAGTTT |
|  | TNNI3 R | CCCGGTTTTCCTTCTCGGTG |
| Actin Alpha Cardiac Muscle 1 | ACTC1 F | GTCGGGACCTCACTGACTAC |
|  | ACTC1 R | CAATTTCACGTTCAGCAGTG |
| Gap Junction Protein Connexin 43 | GJA1 F | TGGTAAGGTGAAAATGCGAGG |
|  | GJA1 R | GCACTCAAGCTGAATCCATAGAT |
| Calcium Voltage-Gated Channel Subunit Alpha1 C | CACNA1C F | TAGGCATTGGGGTGAAAGAG |
|  | CACNA1C R | GAAGATGATTCCAACGCCAC |
| Sodium Voltage-Gated Channel Alpha Subunit 5 | SCN5A F | TCTCTATGGCAATCCACCCCA |
|  | SCN5A F | GAGGACATACAAGGCGTTGGT |
| Potassium Voltage-Gated Channel Subfamily Q Member 1 | KCNQ1 F | GACTCTGCTGACACCCATCA |
|  | KCNQ1 R | ACAAAGTACTGCATGCGTCG |
| Ribosomal Protein L32 | RPL32 F | AGGCATTGACAACAGGGTTC |
|  | RPL32 R | GACGTTGTGGACCAGGAACT |
| Ryanodine Receptor 2 | RyR2 F | GCGAAGACGAGATCCAGTTC |
|  | RyR2 R | CTGTTGCCAAATCCTTCTGC |
| NADH: Ubiquinone Oxidoreductase Subunit V3 | NDUFV3 F | CTGAGCCGTTTGACAACACTA |
|  | NDUFV3 R | TGAGGTCTAAGAAGGTGTACGTG |
| Oxoglutarate Dehydrogenase | OGDH F | GGGCGTGGTATATGAGACCTT |
|  | OGDH R | TGTGGTGAATCCAATCTGGTTG |
| Mitochondrially Encoded Cytochrome C Oxidase III | COX3 F | TTGCTCTGACCGAATTAAGCC |
|  | COX3 R | CTCAGTGGCAGGATCGTAGT |
| Cytochrome C Oxidase Subunit 5B | COX5B F | TGTGAAGAGGACAATACCAGCG |
|  | COX5B R | CCAGCTTGTAATGGGCTCCAC |

Table S3: Antibodies list used for Western Blot, flow cytometry and fluorescent Immunohistochemistry experiments.

| **Antibody host and target** | **Reference** |
| --- | --- |
| Rabbit anti-cardiac Troponin T | Abcam ab45932 |
| Mouse anti-ATP5A | Abcam ab14748, USA |
| Rabbit anti-tom20 | Santa Cruz sc-11415, FL-145, USA |
| Mouse anti-α-actinin | Merck A7811, USA |
| AlexaFluor goat anti-rabbit 568 | Invitrogen A11036 |
| AlexaFluor goat anti-mouse 488 | Invitrogen A11029 |
| Hoechst 33342 | Invitrogen H3570 |


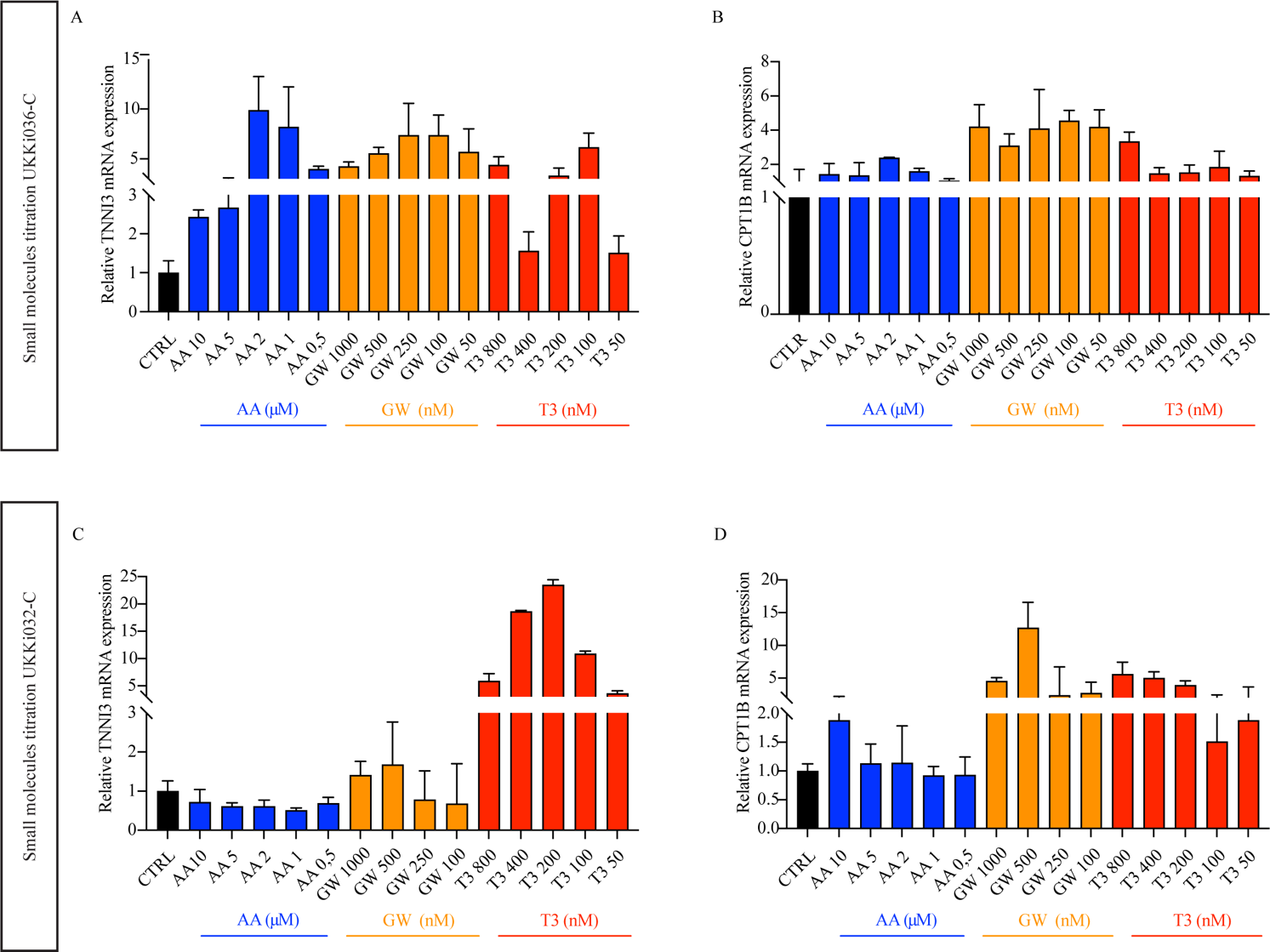


**Figure S2:** Cell line dependent small molecules dose titration. iPSC-line-dependent dose response-effect on CMs at day 27 of differentiation (Fig. 2). For further experiments, the cell clone-dependent optimal concentrations (UKKi036-C: AA 2 and 1 μM, GW: 250 and 100 nM; and T3: 200, and 100 nM; UKKi032-C: AA 10 and 5 μM, GW: 1000 and 500 nM; and T3: 400, and 200 nM; UKKi037-C AA 10 and 5 μM, GW: 1000 and 500 nM; and T3 800 and 400 nM – Fig. S2) were used.


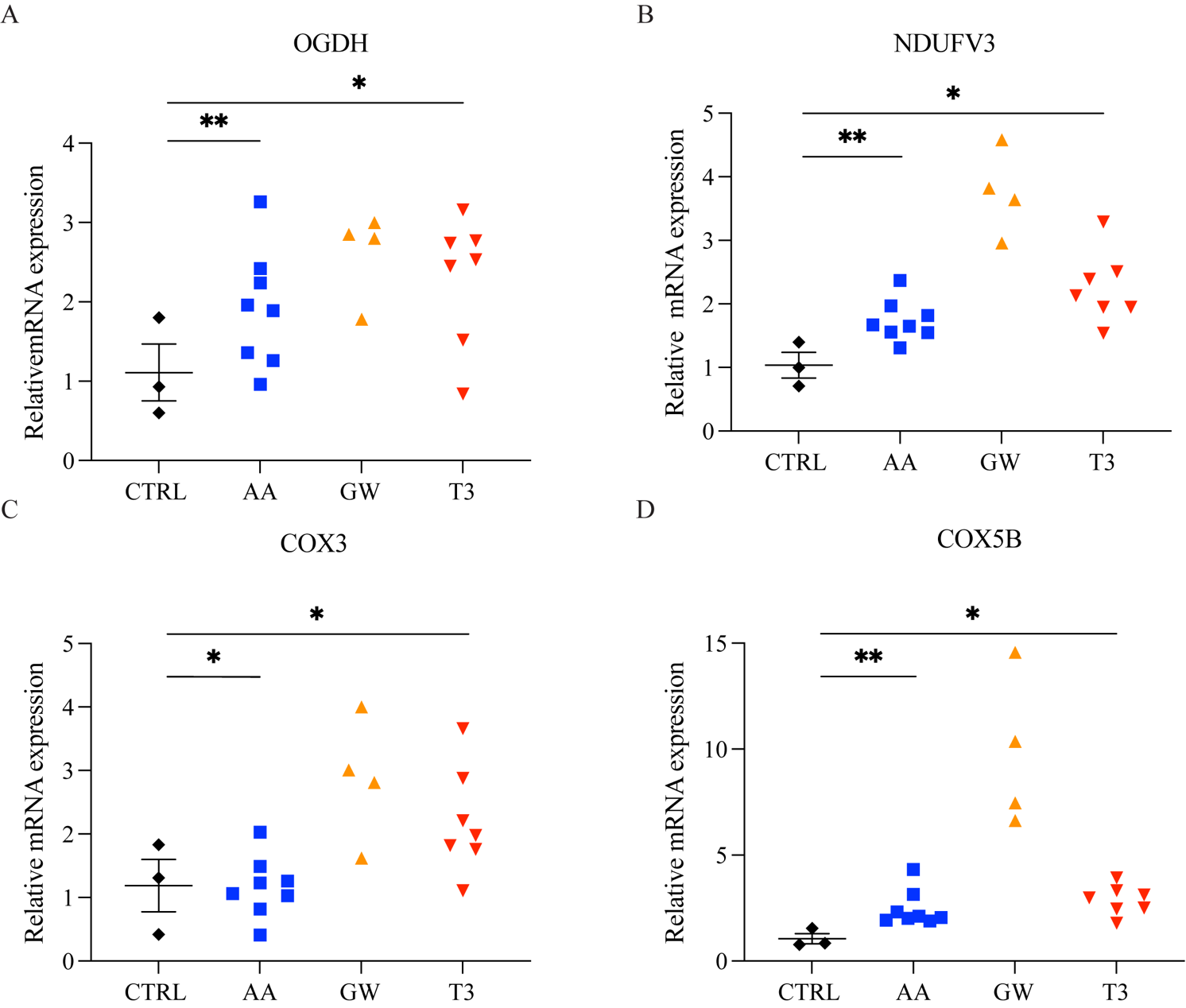


**Figure S3:** AA and GW treatment enhances levels of mitochondrial key enzymes expression. a) OGDH CTRL versus AA, GW, and T3, respectively: 1.110 (0.6000 – 1.800) versus 1.919 (1.285 - 2.375; *p= 0.0078*) versus 2.608 (2.035 - 2.963. *n.s.*) versus 2.287 (1.520 - 2.770; *p=0.0156*); b) NDUFV3 CTRL versus AA, GW, and T3, respectively: 1.037 (0.7100 – 1.400;) versus 1.738 (1,553 – 1.933, *p=0,0078*), 3.750 (1.738 – 4.390; *n.s.*). and 2.251 (1.950 – 2.510; *p=0.0156*); c) COX3 CTRL versus AA. GW. and T3. respectively: 1.187 (0.4200 - 1.830) versus 1.166 (0.8725 - 1.433; *p= 0.0234*) versus 2.860 (1.918 - 3.753; *p=n.s.)* versus 2.203 (1.760 - 2.880; *p= 0.0156*); d) COX5 CTRL versus AA. GW. and T3. respectively: 1.053 (0.7800 - 1.540) versus 2.471 (1.950 - 2.943; *p= 0.0078*) versus 9.758 (6.838 - 13.52; *p=n.s.)* versus 2.873 (2.460 - 3.320*; p= 0.0156*)


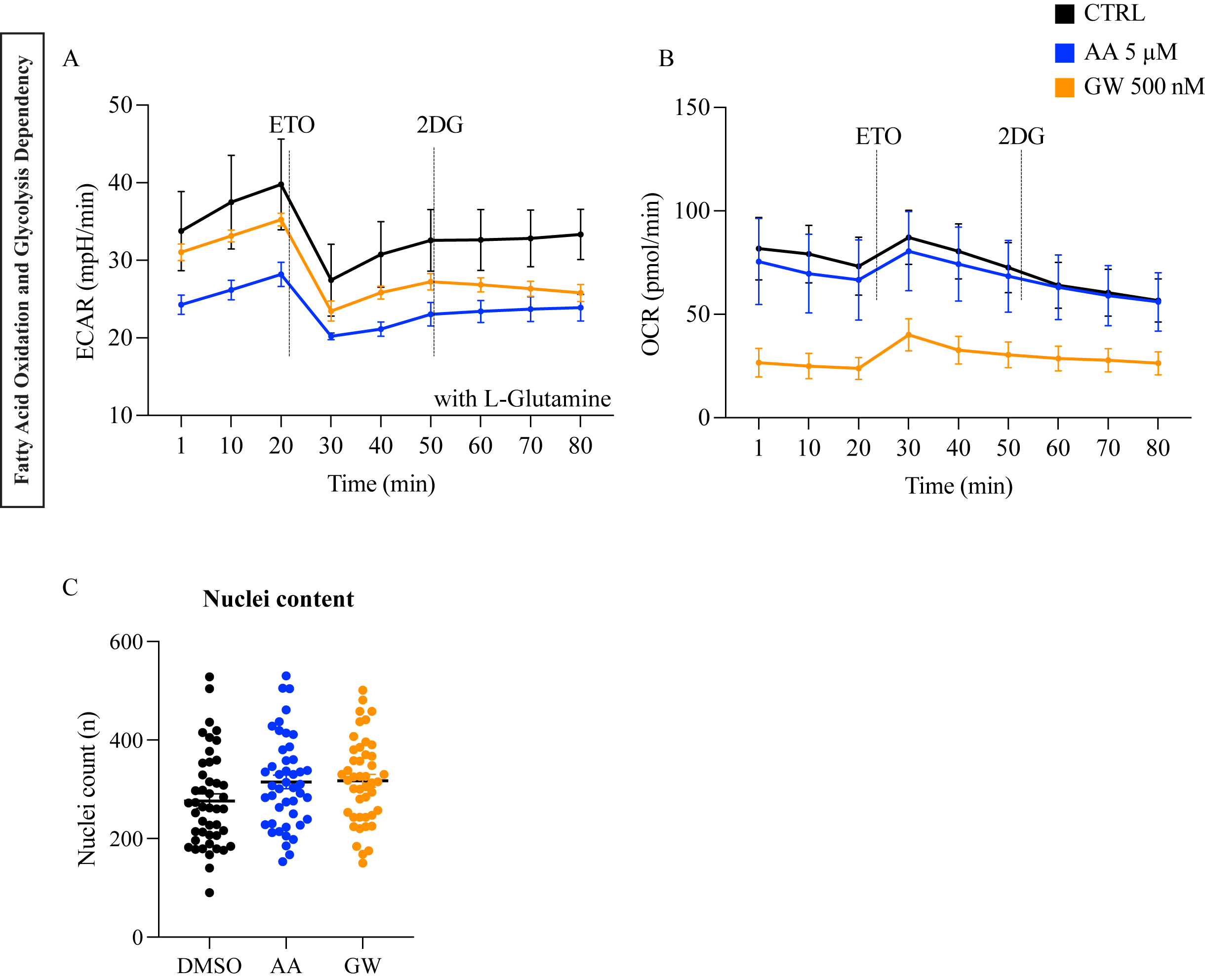


Figure S4: Experiments were performed using day 27 iPSC-CMs line UKKi036-C. (a) Raw extracellular acidification rate (ECAR) of iPSC-CMs cultured in the seahorse medium with L-glutamine. Addition of ETO and 2DG block the FAO and glucose-dependent glycolysis respectively. (b) Raw oxygen consumption rate (OCR) in cells at day 27 of differentiation after sequential administration of ETO and 2DG.

Table S4: Relative DNA content per single nucleus measured on three independent experiments with iPSC lines: UKKi036-C UKKi032-C and UKKi037-C combined n=6-12.

|  | 2N | | | >2N | | | >4N | | |
| --- | --- | --- | --- | --- | --- | --- | --- | --- | --- |
|  | **Mean** | **SD** | **N** | **Mean** | **SD** | **N** | **Mean** | **SD** | **N** |
| **DMSO** | 100 | 002 | 900 | 100 | 015 | 900 | 100 | 042 | 600 |
| **AA** | 100 | 004 | 1800 | 106 | 029 | 1800 | 216 | 179 | 1200 |
| **GW** | 102 | 002 | 1800 | 090 | 018 | 1800 | 148 | 107 | 1200 |
| **T3** | 102 | 004 | 1800 | 095 | 027 | 1800 | 106 | 106 | 1200 |


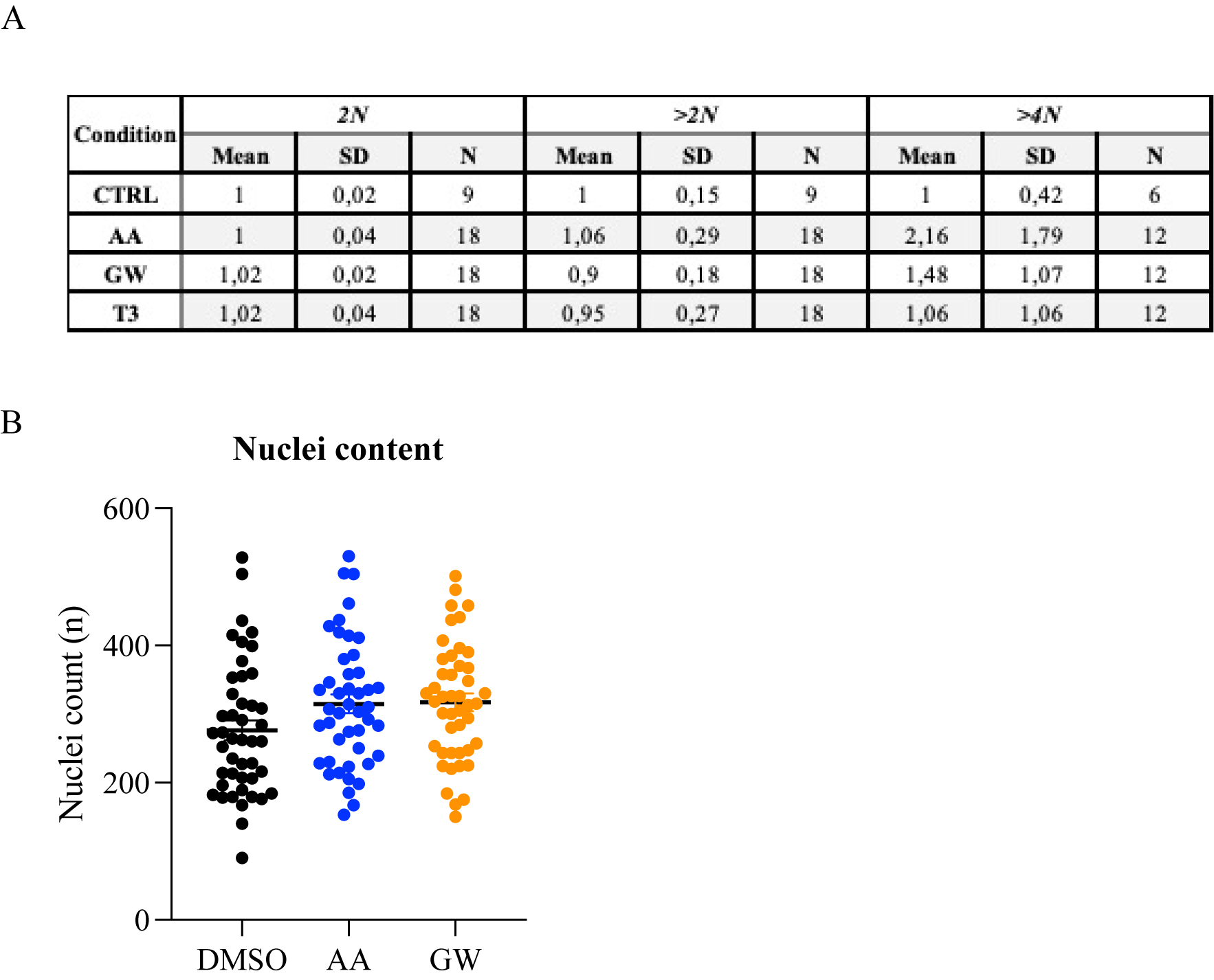


**Figure S5:** Cell nuclei count per well measured by Hoechst staining and 20X magnification imaging using the Evos microscope and ImageJ show no significant changes in number of nuclei present.


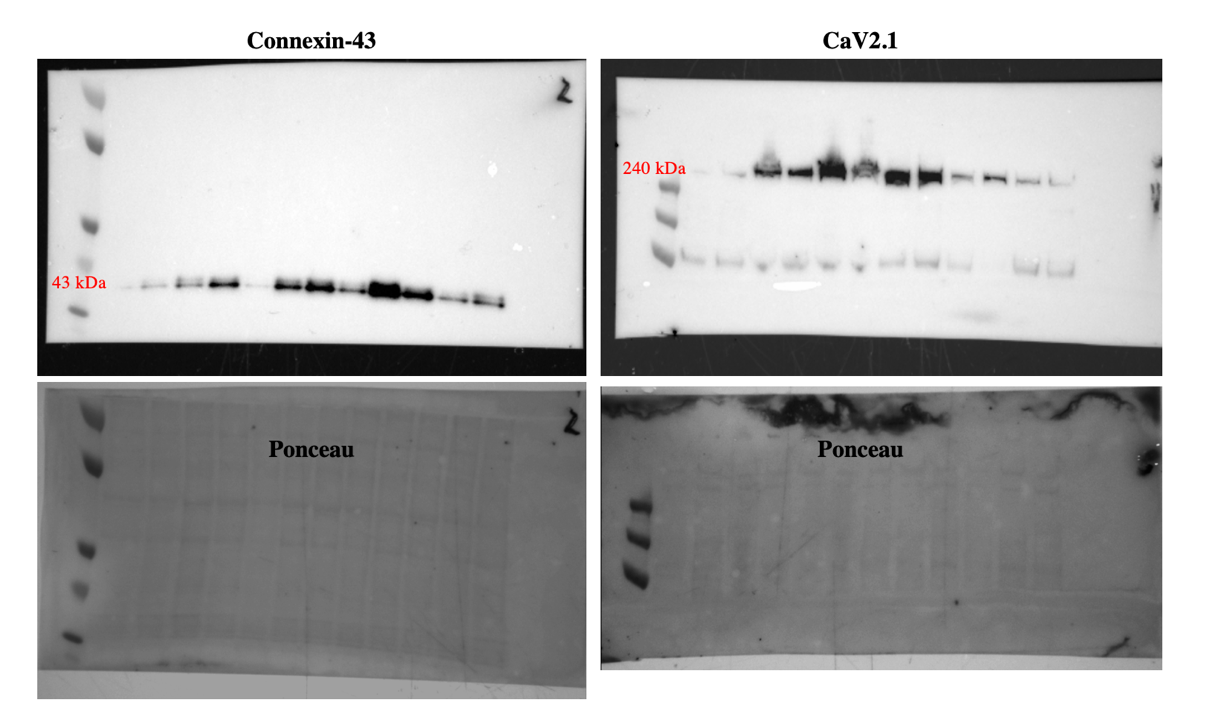


**Figure S6:** Full-length blots of connexin-43 (left) and CaV1.2 (right) with marker and molecular weight of protein of interest in red. Western blot**s** were performed on pooled proteins from all three cell lines. Equal amount of protein was loaded in each lane. Ponceau staining is shown underneath the Western blot panel as a loading control.
